# Giant nanomechanical energy storage capacity in twisted single-walled carbon nanotube ropes

**DOI:** 10.1038/s41565-024-01645-x

**Published:** 2024-04-16

**Authors:** Shigenori Utsumi, Sanjeev Kumar Ujjain, Satoshi Takahashi, Ryo Shimodomae, Tae Yamaura, Ryosuke Okuda, Ryuichiro Kobayashi, Oga Takahashi, Satoshi Miyazono, Naoki Kato, Keiichi Aburamoto, Yuta Hosoi, Preety Ahuja, Ayumi Furuse, Yuma Kawamata, Hayato Otsuka, Kazunori Fujisawa, Takuya Hayashi, David Tománek, Katsumi Kaneko

**Affiliations:** 1 Department of Mechanical and Electrical Engineering, Faculty of Engineering, Suwa University of Science, Chino, Japan; 2 Research Initiative for Supra-Materials, Shinshu University, Nagano, Japan; 3 Center for Advanced Sensor Technology, University of Maryland Baltimore County, Baltimore, MD USA; 4 Department of Science and Technology, Interdisciplinary Graduate School of Science and Technology, Shinshu University, Nagano, Japan; 5 Department of Water Environment and Civil Engineering, Shinshu University, Nagano, Japan; 6 Physics and Astronomy Department, Michigan State University, East Lansing, MI USA; 7 Department of Physics, University of Johannesburg, Johannesburg, South Africa

**Keywords:** Carbon nanotubes and fullerenes, Devices for energy harvesting

## Abstract

A sustainable society requires high-energy storage devices characterized by lightness, compactness, a long life and superior safety, surpassing current battery and supercapacitor technologies. Single-walled carbon nanotubes (SWCNTs), which typically exhibit great toughness, have emerged as promising candidates for innovative energy storage solutions. Here we produced SWCNT ropes wrapped in thermoplastic polyurethane elastomers, and demonstrated experimentally that a twisted rope composed of these SWCNTs possesses the remarkable ability to reversibly store nanomechanical energy. Notably, the gravimetric energy density of these twisted ropes reaches up to 2.1 MJ kg^−1^, exceeding the energy storage capacity of mechanical steel springs by over four orders of magnitude and surpassing advanced lithium-ion batteries by a factor of three. In contrast to chemical and electrochemical energy carriers, the nanomechanical energy stored in a twisted SWCNT rope is safe even in hostile environments. This energy does not deplete over time and is accessible at temperatures ranging from −60 to +100 °C.

## Abstract (web summary)

A single-walled carbon nanotube spring stores three times more mechanical energy than a lithium-ion battery, while offering wide temperature stability and posing no explosion risk.

## Main

**Fig. 1:**
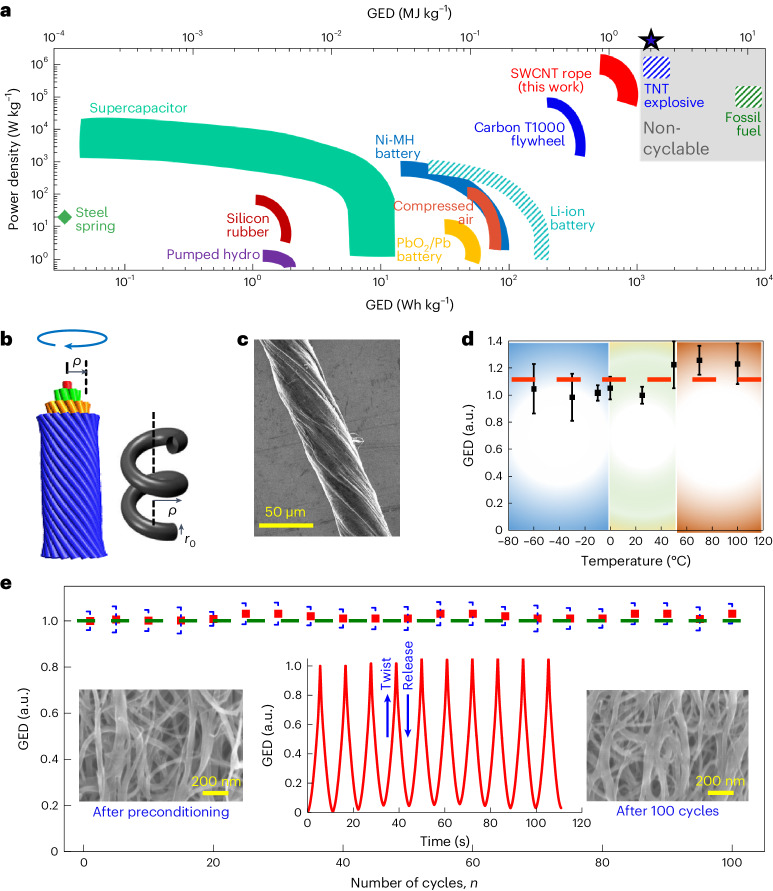
Performance of a twisted SWCNT rope and other viable energy carriers. **a**, Energy-storage and power-density ranges of common energy storage media. Hatched areas (LIB, TNT explosive and fossil fuel) identify potentially unsafe carriers of electrochemical or chemical energy that may catch on fire or explode in hostile environments. Unlike in other energy carriers, release of record energy amounts stored in chemical bonds of fossil fuels and explosives is irreversible. ★, Power density of TNT explosive, ∼6.2 × 10^11^ W kg^−1^. We graphically distinguished non-cyclable from cyclable storage media and identified potential safety risks. **b**, Schematic morphology of a twisted SWCNT rope (left) and a single constituent strand (right), reproduced from ref. ^16^, © American Physical Society. **c**, SEM micrograph of a twisted SWCNT rope. **d**, Temperature dependence of the maximum GED of a twisted SWCNT rope at torsional strain *ε* ≈ 0.6. The maximum GED has been normalized with respect to the GED at 25 °C. Individual data points are depicted as black solid squares; data are presented as the mean ± s.d. for *n* = 3 y-rope (TPU) samples. During a continuous measurement, each rope sample was first heated from 298 K to high temperature and subsequently cooled to low temperature. At each temperature, three consecutive twist/release cycles were performed. The dashed orange line suggests that, within error bars, the GED is rather independent of temperature. **e**, Cycling stability of a y-rope (TPU) during 100 consecutive twist/release cycles. The normalized GED is measured up to a maximum torsional strain *ε* = 0.6 at a rotational frequency of 110 rpm. The time dependence of the GED during the first few cycles is shown in the inset. Individual data points are depicted as red solid squares; data are presented as the mean ± s.d. for *n* = 3 y-rope (TPU) samples. Insets: SEM micrographs of *y*-rope (TPU) after initial preconditioning cycles and after 100 twist/release cycles.

Single-walled carbon nanotubes (SWCNTs) offer unique possibilities to produce high-performance energy-conversion and energy storage devices, such as solar cells, batteries or supercapacitors^1^, and numerous technical challenges affecting SWCNTs have been overcome since their discovery in 1993^2,3^. Developing technologies that can meet the net-zero objective and next-generation needs requires multiple energy sources. Energy acquisition in itself is not enough, and an environmentally compatible approach for efficient energy storage during times of high demand is presently the top-ranking priority for humankind. The currently used reversible energy storage mechanisms include electrochemical potential energy in batteries and capacitors, gravitational potential energy in elevated water reservoirs, and mechanical energy. Large amounts of energy can also be stored reversibly with ∼98% retrieval efficiency in superconducting magnets; however, this approach suffers from an extremely high refrigeration cost^4–6^. Mechanical energy can be stored statically in conventional mechanical springs made of steel, but displays a low gravimetric energy density (GED) of ∼1.4 × 10^−4^ MJ kg^−1^ (ref. ^7^). Less compact mechanisms for reversibly storing high energy densities include pumped hydroelectricity, compressed gases and carbon T1000 flywheels^8^. Electrochemical energy storage devices are more compact. The market for cyclable electrochemical energy storage is dominated by lithium-ion batteries (LIBs)^9^, which display GED values ≤0.72 MJ kg^−1^, four orders of magnitude higher than mechanical springs. However, the capability to store high energy densities typically results in safety risks. Cyclable electrochemical energy carriers, including LIBs, may catch fire in a hostile environment in a manner similar to non-cyclable storage media, including fossil fuels and explosives. A comparison of currently available energy storage media is shown in Fig. 1a. Only a few of these media and mechanisms are suitable for storing and delivering energy across a wide range of temperatures in remotely miniaturized sensors or medical implant devices. As discussed below, ropes of twisted SWCNTs exhibiting elevated nanomechanical energy and retaining stability across a wide temperature range may be suitable for such applications.

Being 100 times stronger and five times stiffer than steel at a fraction of its weight, SWCNTs are known for their unparalleled mechanical toughness. They combine a high Young’s modulus of 1 TPa with a tensile strength exceeding 100 GPa and an elastic strain limit of up to 20–30% (refs. ^10–13^). Moreover, twisted CNT ropes can rapidly release stored mechanical energy^14^. Because the mechanical behaviour of graphitic carbon, the constituent of CNTs, does not change much below its melting point, the stored energy should not deplete over time and, unlike in alternative energy storage systems, should be accessible at temperatures ranging from cryogenic to a few thousand kelvin. Theoretical studies suggested the possibility to store exceptional amounts of energy in twisted SWCNT ropes^15–17^ with GED values ≤8 MJ kg^−1^; in particular, mechanical energy storage with SWCNTs is promising^7^. Although nanomechanical energy storage in ultralong triple-walled CNTs^8^, multiwalled (MW) CNT fibres^7,18^, MWCNT/graphene composites^19^ and MWCNT ropes has been previously studied, the degree to which CNT systems may be competitive with alternative energy storage media remains unclear. Unlike a bundle of carbon fibres consisting of irregular graphitic nanoribbons that store energy during stretching, four different channels store energy in a twisted SWCNT rope^15–17^. When the rope is twisted, each strand is subjected to stretching, twisting, compression and bending. Simultaneously, the stretched outermost mantle of the rope subjects the interior to a very high hydrostatic pressure. Depending on the diameter, each channel contributes a comparable amount toward the GED of a twisted SWCNT rope^15–17^ which acts as a torsional spring. A recent study by Baughman and co-workers highlighted the efficient generation of mechanical energy through the stretching and twisting of plied triple-MWCNT yarns in an HCl aqueous solution. The resulting maximum output GED reached 0.401 × 10^−3^ MJ kg^−1^, although it was not notably substantial^20^. The lower GED of MWCNTs is a consequence of their relatively low elastic moduli and tensile strengths, compared with SWCNTs.

The observed energy storage densities achieved using MWCNTs were significantly lower than previously predicted theoretical values. This discrepancy suggests that the potential of individual CNTs has not yet been fully exploited. Consequently, the objective of this study is to experimentally demonstrate the remarkable energy storage potential of SWCNTs subjected to twisting. This approach capitalizes on the exceptional elasticity and lightweight nature of SWCNTs and aims to reveal their capacity to store substantial amounts of nanomechanical energy. In the present article, we provide experimental evidence that a twisted SWCNT rope, fabricated under microwave irradiation using thermoplastic polyurethane (TPU) as modifier (hereafter named y-rope (TPU)), can directly store abundant mechanical energy safely and reversibly, with a excellent GED value of ≤2.1 ± 0.07 MJ kg^−1^ at a power density of ≤1.85 ± 0.43 MW kg^−1^. A schematic of the morphology of the twisted SWCNT rope and one of its SWCNT strands is shown in Fig. 1b. In a realistic rope, each strand represents a yarn containing numerous nanotubes. A scanning electron microscopy (SEM) micrograph of a SWCNT rope with a diameter of 45 µm, exhibiting uniform twist along its length with elevated and lowered wave-like ridges propagating through the surface of the SWCNT ropes in a spiral direction, is presented in Fig. 1c. As shown in Fig. 1d, the maximum GED value was independent of temperature. Their wide useful temperature range and superior power density of these ropes provide significant advantages over less safe electrochemical media, including LIBs.

The degree of nanomechanical energy recovery efficiency between load cycles is equally important, as shown in Fig. 1e. The GED curves demonstrated highly symmetric profiles during the subsequent twist/release cycles, as shown in the inset, after a few preconditioning cycles. The energy recovery ratio or energy density between subsequent cycles was almost unity, even after 100 cycles. Such preconditioning by load cycling is required and is well established in polymer composites^21^. During the initial loading cycles, a permanent structural change was caused in the y-rope (TPU) as clearly seen by comparing the SEM micrographs in Supplementary Fig. 1. The polymer film, which initially covered the surface of the y-rope (TPU) as a cladding, was removed during the initial preconditioning cycles as the individual tubes straightened within the rope under strain. This can be seen from the SEM micrographs after the preconditioning cycles and after 100 cycles, as shown in the inset of Fig. 1e. The SWCNT rope strengthened as more SWCNTs in the core were aligned and contributed to bearing the load.

The physical properties of suitable SWCNT ropes depend on their fabrication process, which we show to be straightforward.

## Mechanically tough SWCNT ropes and their characterization

Starting from commercially available materials containing SWCNTs with a diameter of 1.5 nm and typical length of 1 μm, we used different fabrication processes to form SWCNT ropes. The details of the SWCNT rope fabrication procedures are provided below in ‘Preparation of SWCNT ropes’, further supplemented by Supplementary Fig. 2. We distinguished between ropes formed by the yarn method (y-rope), roll method (r-rope) and dispersion method (d-rope). The high-resolution transmission electron microscopy (HRTEM) micrographs and Raman spectrum of the initial SWCNT material shown in Supplementary Figs. 3 and 4, respectively, served as a qualitative indicator of the graphitic perfection of the CNTs.

**Fig. 2:**
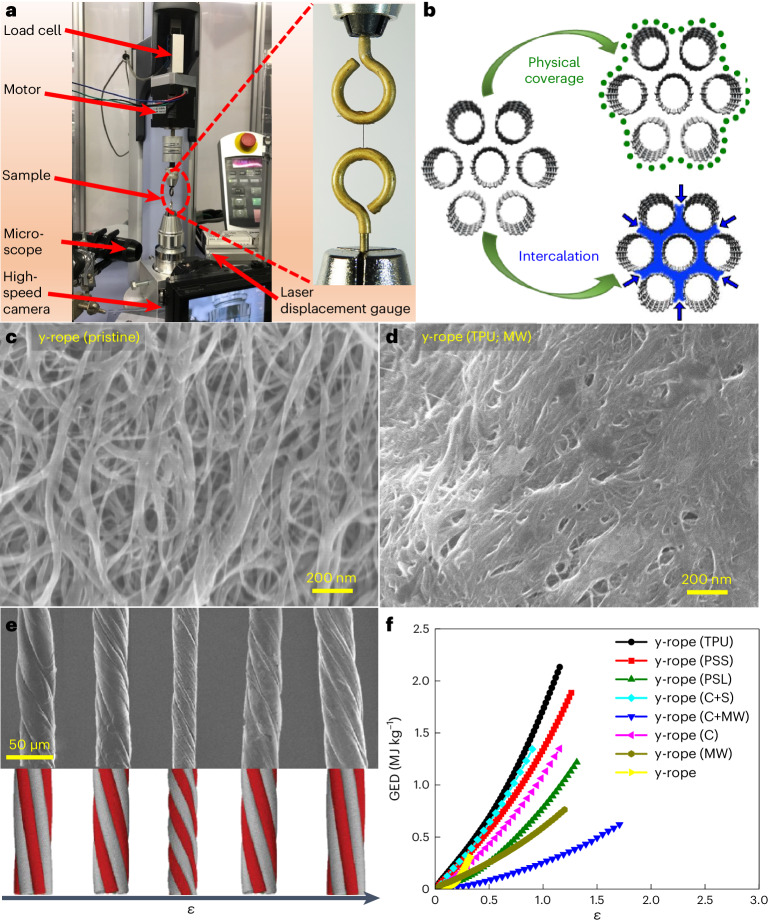
Equipment and measurement of the GED of twisted SWCNT ropes. **a**, Instrumentation used for dynamic GED measurements of twisted SWCNT ropes. Inset: magnified view of eye hooks used to mount the rope sample. **b**, Schematic representation of functionalization processes used to deposit carbon or sulfur onto the surface or intercalate polymers into the SWCNT yarns. **c**,**d**, SEM micrographs of y-ropes in their pristine state (**c**) and following deposition of TPU (**d**), followed by microwave (MW) irradiation. **e**, SEM micrographs of y-ropes subjected to different twist strains, compared to the calculated morphology of twisted nanotube ropes (reproduced from ref. ^17^), © American Physical Society). **f**, GED as a function of the torsional strain *ε* in y-rope and modified y-rope samples of comparable diameter (30 ± 4 µm).

Because our main objective was to reversibly store large amounts of energy in a thin twisted fibre, we first needed to identify a reliable measurement technique for this purpose. The instrumentation used for dynamic GED measurements is shown in Fig. 2a. At the core of the rather complex instrument, which is further discussed in Methods, two clamps hold onto the opposite ends of an SWCNT rope sample of known mass *m*. The upper clamp, rotating about the rope axis, was driven by a stepper motor, and was connected to a gauge that measured the axial force (Supplementary Video 1). The lower clamp was connected to a gauge that measured the torque exerted by the strained rope. The energy *E* stored in the rope was obtained by integrating the force and torque, and GED = *E/m*.

To quantitatively characterize torsion in a twisted SWCNT rope, we first defined the torsional strain^15–17^ as *ε* = *φD*/*2L*_0_, where *D* is the rope diameter, *L*_0_ is the length of the straight rope corresponding to the distance between the eye hooks, and *φ* is the twist angle in radians. To reversibly store energy, the deformation must remain within the elastic limit of the effective torsional spring representing the rope, setting an upper limit for *ε*.

Supplementary Fig. 5 shows significant differences in terms of maximum GED values and graphitic perfection, depending on the fabrication method used. The yarn method yields the highest GED values, with an average value 〈GED〉 = 0.22 ± 0.05 MJ kg^−1^ at an average twist value 〈*ε*〉 = 0.95. With no further processing, the GED values of y-rope, r-rope and d-rope are rather low. A low GED may be partially related to tube bundling. The tube bundling refers to the aggregation of multiple SWCNTs into ropes and can induce strain and lattice distortions due to intertube interactions, which can serve as sites for the introduction of defects and disorders, resulting in a lower GED. We gained insights into the influence of bundle morphology on the energy storage performance of the SWCNT ropes through the SEM micrographs (Supplementary Fig. 5b). Evidently, the d-rope, characterized by the smallest average bundle size, exhibited the lowest GED. Conversely, as the bundle size of y-rope increased, the GED also increased. All these factors—low packing density, high tortuosity and the tube bundling effect—impede efficient nanotube-to-nanotube load transfer, resulting in inferior strength and stiffness, and ultimately a low GED value. This observation is substantiated by examining the linear density of the prepared ropes, revealing a discernible decrease in the GED with an increase in linear density (Supplementary Fig. 6).

## SWCNT rope reinforcement processing

To address the aforementioned limitations, a polymer treatment was designed to enhance inter-SWCNT load transfer while preserving the beneficial mechanical properties of individual nanotubes. This approach accelerates the elastic deformation of individual SWCNTs, thereby enhancing the performance of SWCNT ropes for energy storage. Because ropes produced by the yarn method appear to be the most promising for energy storage, we modified the SWCNT y-rope material with polymers, including TPU, short-polystyrene (PSS), long-polystyrene (PSL) and poly(vinyl alcohol) (PVA). Thermoplastic polymers are elastic and melt-processable and are well known to act as superplasticizers that enhance the stretchability, flexibility and durability of carbon composites^22,23^. Modification of the SWCNT y-rope by the intercalation of polymers or deposition of either carbon or sulfur, followed by microwave irradiation, is depicted schematically in Fig. 2b. The changes in the morphology of the SWCNT ropes caused by polymer modification and carbon or sulfur deposition are shown in Supplementary Figs. 7 and 8. SEM micrographs of the SWCNT ropes prior to processing and following modification with TPU, shown in Fig. 2c,d, respectively, indicate that the overall morphology changed completely upon modification. Further, the effect of microwave irradiation can be observed from the SEM micrographs, which display very crucial differences between the surface and interstitial site of the TPU-wrapped SWCNTs strands before and following microwave irradiation (Supplementary Fig. 7). The micrograph shows uniform wrapping of TPU over y-rope fibres, with no significant difference between their surface and interstitial sites. However, after irradiation, molten TPU diffused through the interstitial sites, decorating the exterior of the SWCNTs and acting as a potential linker for the adjacent SWCNT and SWCNT strands (increasing the close packing of the y-rope (TPU)).

The uniformity of the modification with TPU stems from the hydrophobicity of this polymer, as evidenced by the cross-sectional HRTEM micrograph shown in the Supplementary Fig. 9. Such closely packed intertube connected ropes may aid uniform load transfer and help retain the mechanical properties of the nanoscale SWCNT rope samples, resulting in a high GED^24^. Other modifications resulted in different morphological changes, as shown in Supplementary Figs. 8 and 10.

Our interpretation of the morphological changes in the SWCNT ropes is supported by the Raman spectra shown in Supplementary Fig. 11 and Supplementary Table 1. The y-rope (TPU) show the most pronounced upshift in the G mode, indicating a strong interaction between the polymer and the SWCNTs^25,26^, with the polymer acting as a potential linker. The G/D ratio of y-rope (TPU) is 84.8, which is slightly smaller than that of y-rope, but much larger than those of carbon- or sulfur-deposited y-rope.

Furthermore, polymer modifications enhanced the mechanical properties of the y-ropes, as confirmed by their stress–strain curves. The Young’s modulus *E*, and the tensile strength *σ*_B_ and elongation *ε*_B_ at the break point of the modified y-ropes are significantly improved compared with the pristine y-rope. In particular, y-rope (TPU) has the largest values of *σ*_B_ and *ε*_B_; and the *E* is larger than that of y-rope (Supplementary Fig. 12 and Supplementary Table 2), resulting in high mechanical energy storage.

SEM micrographs of y-ropes (TPU) subjected to different twist strains are shown in Fig. 2e. The micrographs show a relatively uniform diameter along the length of the pristine rope. Mechanical energy density values achievable using twisted y-ropes and modified y-ropes with comparable diameters 30 ± 4 µm are compared in Fig. 2f. The corresponding torque generated during torsional strain is presented in Supplementary Fig. 13. We observed the highest GED value of 2.1 ± 0.07 MJ kg^−1^ at torsional strain *ε* ≈ 1.2 and an average GED value of 1.38 ± 0.48 MJ kg^−1^ in y-rope (TPU), which has the narrowest diameter of 30 μm. As expected, the highest energy storage occurred in the narrowest ropes^16^.

The deformation of the SWCNT ropes under pressure was monitored by in situ Raman spectroscopy. Twist cycling improved the alignment of the SWCNTs in the rope and the intertube load transfer. As seen in Supplementary Fig. 14a, we observed an initial upshift in the G band of the y-rope (TPU) during the first ten cycles. Additionally, the Raman spectra also revealed a reversible upshift of the G peak by 3.2 ± 0.2 cm^−1^ while a rope is being twisted (Supplementary Fig. 14b). This shift results from an improved intertube load transfer, causing radial pressure in the interior of the rope and leading to elastic distortion within the tube structure of the individual SWCNTs. Compression of carbon–carbon bonds into the anharmonic regime under pressures of tens of GPa is expected to cause such hardening of vibration modes^17^.

**Fig. 3:**
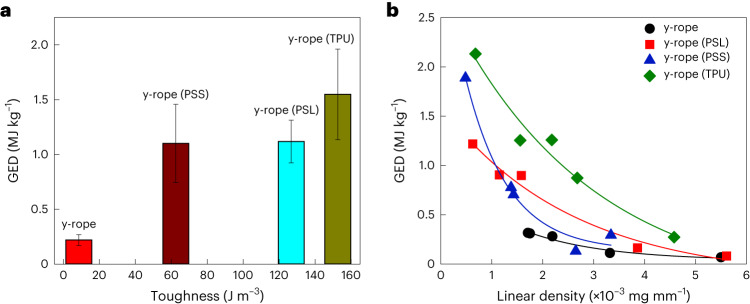
GED as a function of toughness and linear density of y-ropes*.* **a**, Comparative GED of y-rope and modified y-rope samples with respect to their toughness. Toughness was derived by integrating stress with respect to strain in the stress–strain curve shown in Supplementary Fig. 12. The data are presented as the mean ± s.d. for *n* = 3 rope samples for each type. Pristine y-rope, characterized by lower toughness, is associated with the lowest GED, whereas y-rope (TPU) exhibits the highest GED. **b**, Comparative GED of y-rope and modified y-rope samples with respect to their linear density. The linear density demonstrates an inverse correlation with GED, as the fabricated ropes exhibit a decline in GED with an increase in their linear density.

Not all chemical treatments increased the GED in the same way. Under comparable conditions, y-ropes modified by PSS (y-ropes (PSS)) and by PSL (y-ropes (PSL)) both display a torsional strain limit *ε* ≈ 1.3, similar to that of their TPU counterpart. However, the maximum GED values in these systems are smaller than that of y-rope (TPU). Moreover, the toughness of these ropes, as assessed from the stress–strain curve and linear density, exhibits a strong correlation with the GED (Fig. 3). The GED of y-ropes (C), produced by depositing carbon on SWCNT ropes, increases with the number of deposition cycles, with a maximum value GED ≈ 1.35 MJ kg^−1^, which was reached after 80 cycles (Supplementary Fig. 15). Subsequent deposition of sulfur, resulting in y-ropes (C+S), has only a negligible effect on increasing the energy storage capacity to ∼1.37 MJ kg^−1^, but reduces the maximum value of the strain limit to *ε* ≈ 0.8. We conclude that even though the deposited carbon and sulfur improve the intertube coupling in the rope, their effect is inferior to that of TPU in enhancing the GED value of the twisted SWCNT ropes.

Furthermore, we studied the effect of the cross-sectional diameter of the prepared y-rope (TPU) on the GED value and torsional strain limit. As shown in Supplementary Fig. 16a, we observed an increase in the GED and a decrease in the torsional strain limit with decreasing diameter of y-ropes (TPU). The reduction in the torsional strain limit of ∼2.3 in 90-μm-wide ropes to ∼1.2 in 30-μm-wide ropes is significant. As the energy component obeys Hooke’s law in the elastic regime, the total nanomechanical strain energy density GED can be expressed as GED = ½(*k*/*m)ε*^2^, where *k* is the elastic constant and *m* is the mass of a particular rope^7,27^. Our results indicate that the GED of y-ropes (TPU) 30–60 μm in diameter is described well by Hooke’s law in the elastic regime. In addition, the 90-μm-wide rope displayed strong anharmonic behaviour, even at moderate torsion strain values, caused by SWCNTs stretching beyond the elastic limit (Supplementary Fig. 17). The other modified y-ropes demonstrated a similar trend of decreasing GED with increasing cross-sectional diameter (Supplementary Fig. 18).

## Twisted y-rope (TPU) efficient energy output and conversion

Furthermore, we investigated the direct energy output from twisted y-ropes (TPU) by the rotation of a load (eye-hook + paddle) attached to it, which is more than 4 × 10^4^ times higher in weight than the weight of the rope sample. The rope sample was first twisted through 10, 20 and 30 rotations using a motor at 110 rpm, after which it was allowed to untwist with the load. We have defined ‘recovery’ Rr as the ability of the twisted (forward rotation) SWCNT rope to return (reverse rotation) to its original, untwisted state after undergoing a specified number of twist cycles. In particular, after ten twisting rotations, the rope untwisted back to approximately 90% of its initial untwisted configuration. This implies that a residual twist remains in the rope after the untwisting process. The presence of a residual twist suggests that there might be some energy dissipation due to internal friction and air resistance, leading to the decay and eventual cessation of the periodic motion in the system, resulting in a slight deviation from complete recovery. The actual stored energy influences the extent to which Rr surpasses 100% and the duration of the periodic motion. Indeed, we observed Rr values exceeding 100% during reverse rotation of y-rope (TPU) samples twisted through 20 and 30 rotations in the forward direction (Supplementary Video 2).

Moreover, we have observed the efficacy of TPU as a linker in y-ropes (TPU), enabling a strain energy recovery of up to 90 ± 2% within a mere 1.1 s. The ultrashort untwisting time translates into a high power density of ≤1.85 ± 0.43 MW kg^−1^. In comparison, the recovery efficiency decreased to 65 ± 5% in the absence of TPU. Furthermore, we observed a reduction in the energy recovery efficiency of up to 20% over 20 h, which was attributed to structural changes in the rope caused by self-discharge due to slippage relaxation that occurs over time. This energy loss was efficiently reduced in the presence of the TPU polymer.

To demonstrate the application and energy conversion efficiency of the stored mechanical energy in the twisted rope samples, we rotated a circular disc 8 × 10^3^ times heavier than that of the y-rope (TPU) using the energy stored in the twisted ropes. The y-rope (TPU) sample was twisted using a mechanical motor, and the circular disc attached to its other end was allowed to rotate by utilizing the energy generated by untwisting the ropes. The disc attained a maximum angular velocity of 164 rad s^−1^ (angular velocity excluding the effect of friction) with recovery rate Rr ≈ 100% and an energy-conversion efficiency of 22% (Supplementary Fig. 19 and Supplementary Video 3). The energy-conversion efficiency was limited by the effect of friction in the present system, and future endeavours should be to develop a minimum-friction system to derive the maximum energy-conversion efficiency.

## Twisted y-ropes (TPU) with aligned SWCNT configurations

**Fig. 4:**
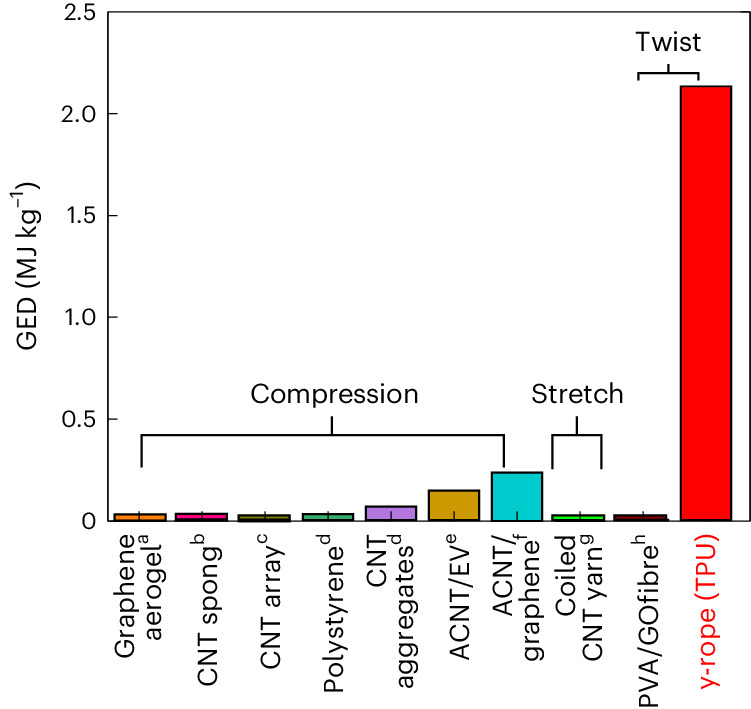
Comparative mechanical GED for various materials. Maximum GED values of different materials subject to compression, stretching or twisting. ^a^Ref. ^31^, ^b^ref. ^30^, ^c^ref. ^28^, ^d^ref. ^35^, ^e^ref. ^33^, ^f^ref. ^19^, ^g^ref. ^34^, ^h^ref. ^29^.

**Fig. 5:**
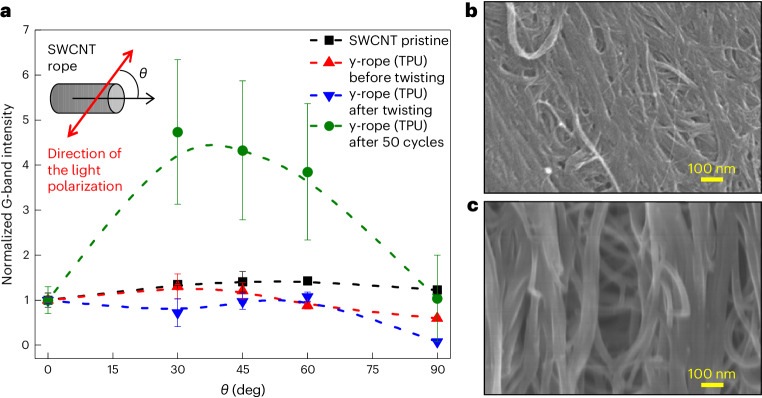
Reorientation of SWCNTs in y-rope (TPU) by twisting. **a**, The normalized G-band intensity of pristine SWCNT and y-rope (TPU) samples as a function of *θ*, the angle between the light-polarization direction and the long axis of the SWCNTs. The G-band intensities are normalized with respect to the intensity at *θ* = 0°. Here, y-rope (TPU) was analysed before twisting to understand the evolution of the orientations during the rope fabrication process. Inset: schematic illustration of the angle between the polarization direction and the SWCNT rope axis. The data are presented as the mean ± s.d. for *n* = 3 samples for each type. **b**,**c**, SEM micrographs of y-ropes (TPU) before (**b**) and after 50 twist/untwist cycles (**c**).

As shown in Fig. 4, we also found that y-ropes (TPU) offered higher GED values than comparable carbon nanocomposites subjected to compressive or tensile stress^19,28–37^. The unusually high energy recovery efficiency of twisted y-ropes (TPU) is significantly higher than values not exceeding 10% in other materials, which also implies a swift dynamic response and excellent performance compared with other mechanical energy storage materials. The high performance of the y-ropes (TPU) may be due to the highly aligned and stable configuration of the SWCNTs, which develops during the rope fabrication process. The alignment of the SWCNTs that evolved during the fabrication of y-rope (TPU) was analysed by polarized Raman spectroscopy and the angular dependence of small-angle X-ray scattering (SAXS) (Supplementary Figs. 20 and 21). Twisting the y-rope (TPU) followed by repeated twist/release cycles resulted in most of the SWCNTs being aligned along *θ* = 30° and 45°, with the distribution centred at *θ* = 37° (Fig. 5a). This finding is further confirmed by the SAXS intensity dependence on the angle of the rope axis to the X-ray beam, which reveals that the intensity of the (10) peak at a measuring angle of 30° after 50 repeated twist/release cycles is notably greater than that of the non-twisted sample. Such an enhanced locally aligned configuration of SWCNTs in the y-rope (TPU) resulted in significant intertube load transfer, and hence high mechanical energy storage. In agreement with the discussion above, SEM micrographs of the y-rope (TPU) before and after 50 twist/untwist cycles (Fig. 5b,c) also clearly illustrate a reduction in slack and elimination of the initial polymer surface cladding as a result of the cyclic loading/unloading process.

The y-ropes (TPU) are distinguished not only by their high energy-conversion efficiency and low self-discharge rates over a very wide temperature range but also offer excellent performance compared with other miniature devices in these respects. Another notable strength of SWCNT ropes lies in their capacity to undergo numerous twist/release cycles. These ropes consistently maintain their GED over at least 450 twist/release cycles (Supplementary Fig. 22), underscoring their remarkable stability. At the same time, twisted y-ropes (TPU) have emerged as a cleaner and safer energy storage medium compared with electrochemical devices used to power nano/microelectromechanical systems devices and wireless respiration sensors that are tolerated by tissues in the human body, an important factor in human healthcare products.

## Potential applications for twisted SWCNT ropes

**Fig. 6:**
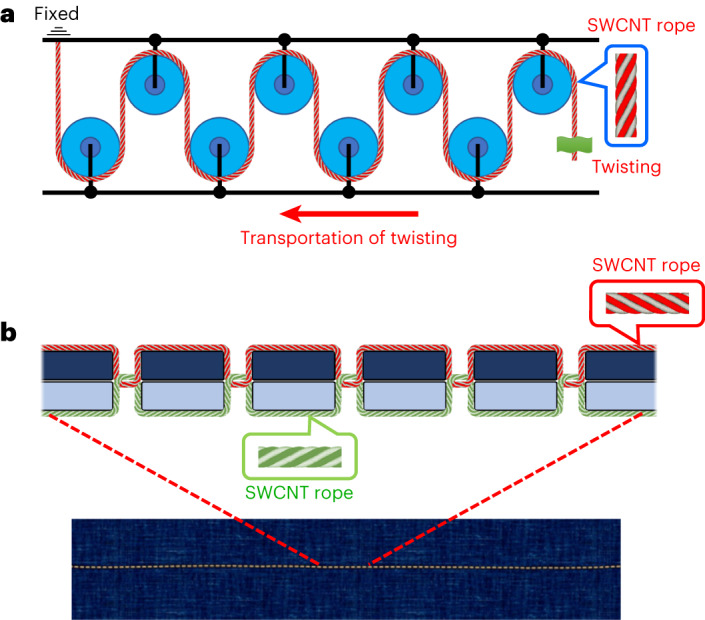
Composite SWCNT pulley model and thread-rich seam model system for massive nanomechanical energy storage. **a**, Composite pulley system with an SWCNT rope. This system efficiently transports twisting motions along the curved SWCNT rope within a compact volume. With one end of the SWCNT rope fixed and the other end free to twist, the twisting motion propagates throughout the entire rope provided the friction between the pulley and the SWCNT rope is minimal. Thus, the composite pulley system makes it possible to produce densely packed SWCNT ropes, optimizing the utilization of twisting energy. **b**, SWCNT-rope-based thread model with seams, which is a highly dense and compact miniature version of the composite pulley system. The composite pulley system can provide a high density of SWCNT ropes in a small volume. When SWCNT ropes are substituted for traditional fibre threads in textile production, the resulting seams resemble a compound pulley system, making it possible to produce highly dense seams with SWCNT ropes. This textile form represents a miniature composite pulley system, offering significantly larger energy storage potential. Seam production is efficiently achieved using a sewing machine. Consequently, a machine analogous to a sewing machine can efficiently produce SWCNT-rope-based textile devices capable of storing substantial mechanical energy within a compact scale. The figure illustrates segments and the textile formed by these segments, with each segment capable of independent twisting to store massive amounts of twisting energy.

Our observation that energy can be stored in a twisted rope is not the first of its kind. Although twisted MWCNT yarns have been reported to store nanomechanical energy reversibly, no quantitative data have been provided on this phenomenon^36^. PVA–graphene oxide nanocomposite fibres were reported to exhibit a temperature-triggered shape memory; however, their gravimetric work capacity remained inferior^37^ to that of silicon rubber (6.6 kJ kg^−1^). Here, we report quantitative data on the amount of energy reversibly stored in a twisted SWCNT rope. The maximum gravimetric energy storage density of the SWCNT ropes is three times greater than that of LIBs, without considering the other advantages of nanomechanical over electrical energy storage. A highly compact and efficient energy storage system—a requisite for future applications—based on twisting of SWCNT ropes can be designed based on composite pulleys or on producing seams with a sewing machine using the regular thread-like properties of CNTs^38^, illustrated in Fig. 6. The composite pulley mechanism makes it possible to produce a highly compact energy storage system by twisting SWCNT ropes (Fig. 6a). This composite pulley system is topologically analogous to using a sewing machine to connect two pieces of fabric with a thread that forms a seam with many stitches (Fig. 6b). Many loose stitches can accommodate very long thread segments in the fabric, each of which can be twisted independently. The threads can be replaced with the SWCNT ropes, making it possible to produce twisting SWCNT ropes–woven textiles that offer massive energy storage. Thus, large amounts of nanomechanical energy can be reversibly stored in a compact volume.

This study demonstrates exceptionally high nanomechanical energy storage, surpassing that of LIBs, in twisted SWCNT ropes. However, longer SWCNT ropes suffer from reduced energy storage capacity, posing a challenge for macroscopic CNT materials^39^ (Supplementary Fig. 23). The SWCNT rope samples investigated in this study were miniscule, restricting their current use to microscale energy storage in hydro and wind power and in small devices. However, advances in SWCNT spinning, especially with thermoplastic elastomers such as TPU as proposed here, offer a promising solution for curing SWCNT ropes. Further research should explore combining this method with better spinning techniques for greater energy storage.

## Conclusions

It is easy to forget that reversible energy storage in personal devices has long been provided dependably by mechanical coil springs in watches and camera shutters. The wind-up torsional spring of SWCNT ropes discussed here is fundamentally no different from the wind-up coil springs of steel, although the convenience and reliability of mechanical energy storage is often overlooked. However, it increases the energy density by four to five orders of magnitude. The nanomechanical system proposed for reversible energy storage has significant advantages over current technologies. The core of the SWCNT rope was functionalized with an elastomer. The energy storage capacity and rate of energy delivery of a rope, which can be reversibly twisted, approaches those of explosives, including gasoline, on a gravimetric basis. The energy storage density of 2.1 MJ kg^−1^ exceeds that of leading electrical or electrochemical energy storage systems, in particular LIBs, by at least a factor of three. In addition, the energy retention rate of a twisted rope exceeds those of competing electrical and electrochemical systems that discharge over time. In contrast, the number of useful charge/discharge or load/unload cycles of the SWCNT rope appeared to be unlimited. Unlike chemical explosives and electrical or electrochemical systems, the storage and delivery of nanomechanical energy in SWCNT ropes is very safe. Although limited ion mobility reduces and even cripples the power delivery of LIBs at very low temperatures, the power density delivered by a twisted SWCNT rope remains rather constant over a wide temperature range, from deep cryogenic temperatures to the boiling point of water. A TPU-impregnated rope of SWCNTs can be manufactured in a straightforward manner at a relatively low cost and is likely to be biocompatible, and thus suitable for medical applications. In particular, these SWCNT systems can be used to develop small power-supply devices for artificial organs that require milliwatts of power to function. Therefore, it should be possible to recharge an SWCNT-rope-based mechanical energy source using the energy created by body movements, and implanted artificial organs powered by such a power source could operate independently over an infinite time without the need for surgical replacement of the power source. Thus, compact textile forms of SWCNT torsional springs can supply the safe energy needed to solve urgent and fundamental issues, and can thereby contribute to increased sustainability.

## Methods

### Materials

CNT samples containing SWCNTs with diameters of 2.0 nm and 1.5 nm, produced by chemical vapour deposition, were procured from MEIJO eDIPS Nano Carbon with the product identification EC2.0 and EC1.5. TPU was procured from BASF Japan, which produces this elastomer under the trade name BASF Elastollan S80A10 TPU. Pellets of short-polystyrene (PSS), with an average molecular weight *M*_w_ ≈ 800–5,000 atomic mass units (a.m.u.), and long-polystyrene (PSL), with an average molecular weight *M*_w_ ≈ 300,000 a.m.u., were both purchased from Polysciences. PVA with an average molecular weight *M*_w_ ≈ 146,000–186,000 a.m.u., and 99+% hydrolysed, was purchased from Sigma-Aldrich. All solvents used in this study were of analytical grade, purchased from Fujifilm Wako Pure Chemical, and used as received. The cyanoacrylate-based adhesive Konishi Bond Alon Alpha Super Jell, used to attach the rope to the instrument for measuring stress, was purchased from Konishi.

### Characterization of the morphology and quality of SWCNT ropes

SEM images of the surface topography of the SWCNT ropes were obtained using a Hitachi High-Technologies Corporation FE-SEM SU8000 series instrument. The microscope was operated at an accelerating voltage of 5 kV under a vacuum of 10^−4^ Pa. SEM was used to determine the morphology of the SWCNTs in the ropes. HRTEM micrographs and cross-sectional images were obtained using a JEOL 2100F electron microscope equipped with a Cs corrector and operated at an accelerating voltage of 80 kV. For cross-sectional HRTEM images, the y-rope (TPU) was cut perpendicular to the long axis using an SEM-FIB (JIB-4610F (JEOL). Raman spectroscopy measurements, performed using a Jasco Laser Raman Spectrometer NRS-4100 with a 532 nm laser, helped us quantify structural changes in the SWCNT rope material. An optical microscope (TBR-1 Yashima Optical) equipped with a Carl Zeiss digital microscope camera (Axiocam ERc 5s) was used to determine the twist angles of the fabricated ropes at an observation magnification of ×400 (eyepiece ×10, objective lens ×40) using a green filter. SAXS experiments were carried out using a thin-film X-ray diffractometer installed at BL8S1 of the Aichi Synchrotron Radiation Center. The incident X-ray wavelength was 0.1355 nm. Taut y-rope (TPU) samples were mounted with clay (UHU patafix) on a silicon non-reflective sample plate.

### Preparation of SWCNT ropes

We found the Meijo eDIPS SWCNTs, which were used in our study, to be highly crystalline, and the amount of disordered carbon was very low, as evident from its high G/D ratio of over 100 in the Raman spectrum shown in Supplementary Fig. 4. SWCNT ropes have been prepared by three methods, namely the yarn method resulting in y-ropes, the roll method yielding r-ropes, and the dispersion method to form d-ropes, and the time sequence of these operations is shown in Supplementary Fig. 2.

In the yarn method for rope preparation, we pulled the longest SWCNT strand from the nanotube agglomerate using tweezers, similar to drawing a thread from a silk cocoon. The samples were weighed and deposited onto Teflon sheets. We further densified the sample by adding a few drops of acetone to each SWCNT strand, which penetrated the intertube and interyarn spaces by capillary action. The elongated sample was subsequently twisted several times manually, resulting in what we call a y-rope.

In the roll method, we first dropped <1 ml of acetone, ethanol or water onto 5–10 mg of SWCNT agglomerate. The film was then sandwiched between Teflon sheets and densified by rolling it normal to the SWCNT direction using a roller that applied mechanical pressure. A thin layer was peeled off from the densified SWCNT sheet using Scotch tape. This layer was cut into thin strips along the direction of the SWCNTs and immersed in toluene. The toluene-soaked strips were individually twisted by hand to form what we call an r-rope.

In the dispersion method, also known as buckypaper, we typically dispersed 1 mg of the SWCNT agglomerate in 50 ml of a solvent, such as acetone, toluene or H_2_O_2,_ and sonicated the suspension. The resulting SWCNT dispersion was filtered and dried at 80 °C to form buckypaper. Similar to the roll method, a thin layer of this buckypaper was peeled off using Scotch tape, cut into strips and immersed in toluene. The toluene-soaked strips were individually twisted manually to form what we call the d-rope.

These fabrication techniques allowed the formation of SWCNT ropes with the desired diameters and lengths to be tested for nanomechanical energy storage using the equipment shown in Fig. 2a. Independent of the fabrication technique, we found that the densification step is crucial for enhancing the load-bearing capacity of the ropes by improving the inter-SWCNT and interyarn load-transfer capabilities^34,35^.

### Modification of SWCNT ropes

The as-obtained SWCNTs ropes were further strengthened by various modification processes, including the deposition of carbon or sulfur or by forming nanocomposites containing TPU or polystyrene (PSS, PSL), followed by microwave irradiation.

To deposit carbon onto the ropes, SWCNT rope samples were placed 25 mm from the carbon rod of a JEOL JEC-530 auto carbon coater equipped with a physical vapour deposition capability. The rod was mounted in a vacuum system between two terminals to provide a high electric current. The deposition of thin carbon films during multiple 10 s cycles, during which the rod was heated to the evaporation temperature of carbon, yielded samples of what we call y-rope (C).

To deposit sulfur, 1 μl of an S/CS_2_ solution (0.05 or 0.5 mg ml^−1^) was placed in a glass tube and then the CS_2_ was completely evaporated. Samples of y-rope (C) were placed in the sulfur-containing glass tube, which was sealed at <1 Pa. Sulfur vapour was then deposited for 1 h under low pressure and at a temperature of 300 °C to form what we call the y-rope (C+S).

To modify SWCNT ropes by TPU, we typically added 100 μl of a TPU/acetone solution (0.54 mg ml^−1^) to the longest SWCNT strands extracted from SWCNT agglomerates. The elongated samples were subsequently twisted manually several times to form ropes during the yarning. Here, it is worth noticing that during all these modification processes, the alignment of the SWCNTs changed significantly (Fig. 5a and Supplementary Figs. 20 and 21). The initial twist angle (*α*) of the prepared rope samples was *α* = 14° ± 4° (Supplementary Fig. 24). Within the s.d. range, the initial twist angle of the prepared samples of comparable dimensions had no significant effect on the overall GED because the rope samples were twisted with a motor in the direction of their initial twist. The resulting samples were maintained under vacuum at 180 °C for 1 h. These ropes were sealed under vacuum (0.06–0.4 Pa) in individual glass tubes, followed by microwave irradiation (200 W) for 5 s, to form SWCNT–TPU nanocomposite ropes called y-ropes (TPU). Although temperature measurement during this irradiation process is difficult, the employed thermocouple must be precisely located near the rope sample. Visual monitoring showed extraneous light which may be due to plasma discharge resulting in a temperature sufficiently higher than the glass-transition temperature of the polymers. PSS- and PSL-based nanocomposite y-ropes (PSS) and y-ropes (PSL) were prepared in a similar way, using PSS/toluene or PSL/toluene solutions (1 mg ml^−1^). The typical rope diameters ranged from 30 to 100 μm, and the rope lengths were 20–30 mm. A PVA-based nanocomposite y-rope (PVA) was prepared using an aqueous solution with the same concentration as the TPU/acetone solution (0.54 mg ml^−1^). PVA powder was dissolved in hot water to form an aqueous solution, out of which 2 μl μg^−1^ was added to the longest SWCNT strands, twisted and dried in a vacuum oven at 100 °C to prepare y-rope (PVA).

### Dynamic measurement of the GED

We measured the energy storage in the SWCNT ropes under torsional strain using a Shimadzu automated testing instrument (EZ Test, EZ-LX) with a maximum load capacity of 500 N, a maximum stroke of 920 mm and a stretching test speed ranging from 0.001 to 1,000 mm min^−1^. To test the sample performance while twisting, the instrument was equipped with eye hooks with a 0.5 mm opening, to which rope samples were mounted firmly using a cyanoacrylate-based adhesive. This adhesive penetrated the interior of the rope, ensuring that all SWCNTs were gripped directly, and no pullout occurred during the load/unload cycles. The tensile force *F* resulting from twisting an SWCNT rope of initial length *L*_0_ and mass *m* was recorded using a Trapezium X data logger.

In parallel, we measured the torque *T* resulting from twisting the SWCNT rope with a minute analogue torque gauge connected to the lower eye-hook and viewed it using a high-speed camera. The torque gauge was monitored using ultrahigh-speed/high-accuracy laser displacement LK-G5000 series LK-Navigator 2 configuration software (Keyence). The experimental set-up, including the measurement instrument, imaging equipment and mounted SWCNT rope sample, is shown in Fig. 2a. During the measurements, we performed a careful analysis of the observed values of *F* and *T*, which were subject to systematic instrument and measurement errors caused by possible slippage between the rope and the mounting eye-hook, and found no significant errors in our data. The rope sample length used in this study was between 20 and 30 mm and the hook-to-hook length was fixed at 5 mm. Notably, the experiments indicated a dependence of the torque on the rope sample length (Supplementary Fig. 23). With the increasing length of the SWCNT-based ropes, their torque, and hence the GED, decreased, which may be associated with macroscopic defects in the SWCNT ropes created during the fabrication processes that deteriorated the mechanical properties of the resulting rope samples.

Our experimental set-up allows us to measure the effective force constant *k*_s_ = *F/*Δ*L* of a given rope, where Δ*L* = *L* − *L*_0_ is the change from the initial rope length *L*_0_. Analogously, we define and measure the effective torque constant of the rope *k*_t_ = *2TL*/*εD*. Assuming that the values of *k*_s_ and *k*_t_ do not change while twisting the rope, we can evaluate GED using the following expression:

1 $${\mathrm{GED}}=1/2[{k}_{\mathrm{s}}\Delta {L}^{2}+{k}_{\mathrm{t}}{\varepsilon }^{2}]/m$$

However, stress relaxation occurs during quasi-static measurements of force and torque, modifying the values of the force and torque constants. As an alternative for the anharmonic regime, we may assume that the force and the torque remain nearly constant between successive turns *n* − 1 and *n*. In this case, we estimate the GED using

2 $${\mathrm{GED}}\approx\mathop{\sum }\limits_{1}^{n}[{F}_{n}\Delta {L}_{n}+\Delta \varphi {T}_{n}]/m$$

where *n* is the number of full turns that increase the total twist angle by Δ*φ* = 2π in radians. *F*_*n*_ is the force and *T*_*n*_ is the torque after *n* turns and Δ*L*_*n*_ = *L*_*n*_ − *L*_*n−*1_ is the length change between turns *n* − 1 and *n*.

However, because both *F*_*n*_ and *T*_*n*_ change continuously, this assumption has a limited value. To compensate for the errors introduced by finite sampling, we replace the summation in equation (2) with integration and obtain:

3 $${\mathrm{GED}}=\left[\int F(\varphi )({\mathrm{d}}L/{\mathrm{d}}\varphi )\delta \varphi +\int T(\varphi )\delta \varphi\right]/m$$

To perform what we call a dynamic measurement, we connected the load cell to a motor rotating at a constant angular velocity and continuously acquired the values of the tensile force *F*(*φ*) and torque *Τ*(*φ*) which depend only on the twist angle *φ*. The integrals extend over the entire range of twist angles *φ* from zero to their maximum. In our measurement, d*L/*d*φ* almost vanishes as the distance between the eyes of the hooks remains the same. In this case, the torque mostly contributes to the GED. Of the three approaches, the one described by equation (3) provides the most accurate estimate of the GED value for a twisted rope. Here, the twist speed had a significant effect on the resulting GED; under comparable conditions, the GED for y-rope was ∼35% higher at 110 rpm compared with that at 10 rpm (Supplementary Fig. 25). This may be attributed to the structural relaxation effect of the SWCNT strands present on the ropes. For a slower rpm, the SWCNT bundles have a sufficiently large time to attain structural relaxation, whereas at a higher rpm, the system does not have sufficient relaxation time, resulting in a 35% enhancement in energy storage. Therefore, all experiments were performed at a twisting speed of 110 rpm, which is the maximum speed at which the rotation number could be counted by lab-made motor equipment and by visual observation.

## Online content

Any methods, additional references, Nature Portfolio reporting summaries, source data, extended data, supplementary information, acknowledgements, peer review information; details of author contributions and competing interests; and statements of data and code availability are available at 10.1038/s41565-024-01645-x.

### Supplementary information

Supplementary Information Supplementary Figs. 1–25 and Tables 1 and 2.

Supplementary Videos 1–3 Supplementary Video 1: Working instrument for dynamic GED measurements of twisted CNT ropes. Supplementary Video 2: Rotation recovery efficiency of y-rope (TPU) untwisting by itself. Supplementary Video S3: Energy conversion efficiency for circular disc rotation using energy stored in twisted ropes.

## Data Availability

All data are available in the main text or in the Supplementary Information and Supplementary Videos.
