# Supplementary material for: Giant nanomechanical energy storage capacity in twisted single-walled carbon nanotube ropes
Source: Nat Nanotechnol. 2024 Apr 16;19(7):1007–15. doi: 10.1038/s41565-024-01645-x (PMC11286531; doi:10.1038/s41565-024-01645-x)
Supplement: Supplementary file 1 — Supplementary Figs. 1–25 and Tables 1 and 2. [file 41565_2024_1645_MOESM1_ESM.pdf]

nature nanotechnology

Article

https://doi.org/10.1038/s41565-024-01645-x

Giant nanomechanical energy storage capacity in twisted single-walled carbon nanotube ropes

In the format provided by the authors and unedited

Table of Contents

Fig. 1. Effect of mechanical twist/release cycling on the single-walled carbon nanotube (SWCNT) rope.

Fig. 2. Fabrication process of a SWCNT rope.

Fig. 3. SWCNT sample used to fabricate SWCNT ropes.

Fig. 4. Raman spectrum of pristine SWCNT.

Fig. 5. Effect of processing method on the gravimetric energy density, tube bundling and the graphitic perfection of SWCNT ropes.

Fig. 6. Effect of processing method on the gravimetric energy density as a function of linear density of SWCNT ropes.

Fig. 7. Effect of microwave irradiation on the TPU-wrapped SWCNT rope.

Fig. 8. Morphology changes in SWCNT y-ropes caused by modification using substances other than TPU.

Fig. 9. HRTEM micrographs of y-rope (TPU).

Fig. 10. SEM micrographs of y-rope (PVA).

Fig. 11. Raman spectra of pristine and functionalized SWCNT ropes.

Fig. 12. Mechanical properties of y-rope samples.

Fig. 13. Torque as a function of the torsional strain ε.

Fig. 14. Structural change during mechanical twist/release cycling.

Fig. 15. Effect of carbon deposition on the maximum GED of SWCNT y-ropes (C).

Fig. 16. GED [MJ/kg] and total stored energy [J] in y-ropes (TPU) of different diameter.

Fig. 17. Morphology change in a y-rope (TPU) at the torsional strain limit.

Fig. 18. GED in modified y-ropes of different diameter.

Fig. 19. Energy conversion efficiency for circular disc rotation.

Fig. 20. Polarized Raman spectra of pristine SWCNT and y-rope (TPU) with relevance to the alignment of SWCNT.

Fig. 21. Synchrotron small-angle X-ray scattering (SAXS) measurement of y-rope (TPU) samples with relevance to the alignment of SWCNT bundle.

Fig. 22. Cycling stability of the y-rope (TPU) during over 450 consecutive twist/release cycles.

Fig. 23. Measurement length dependence of SWCNT rope on torque.

Fig. 24. Twist angle determination during rope sample fabrication.

Fig. 25. Twist speed dependence of GED.

Table 1. G-mode frequencies in pristine and modified SWCNT ropes.

Table 2. The mechanical properties of the y-rope and polymer modified y-ropes, evaluated from their stress vs strain curves.

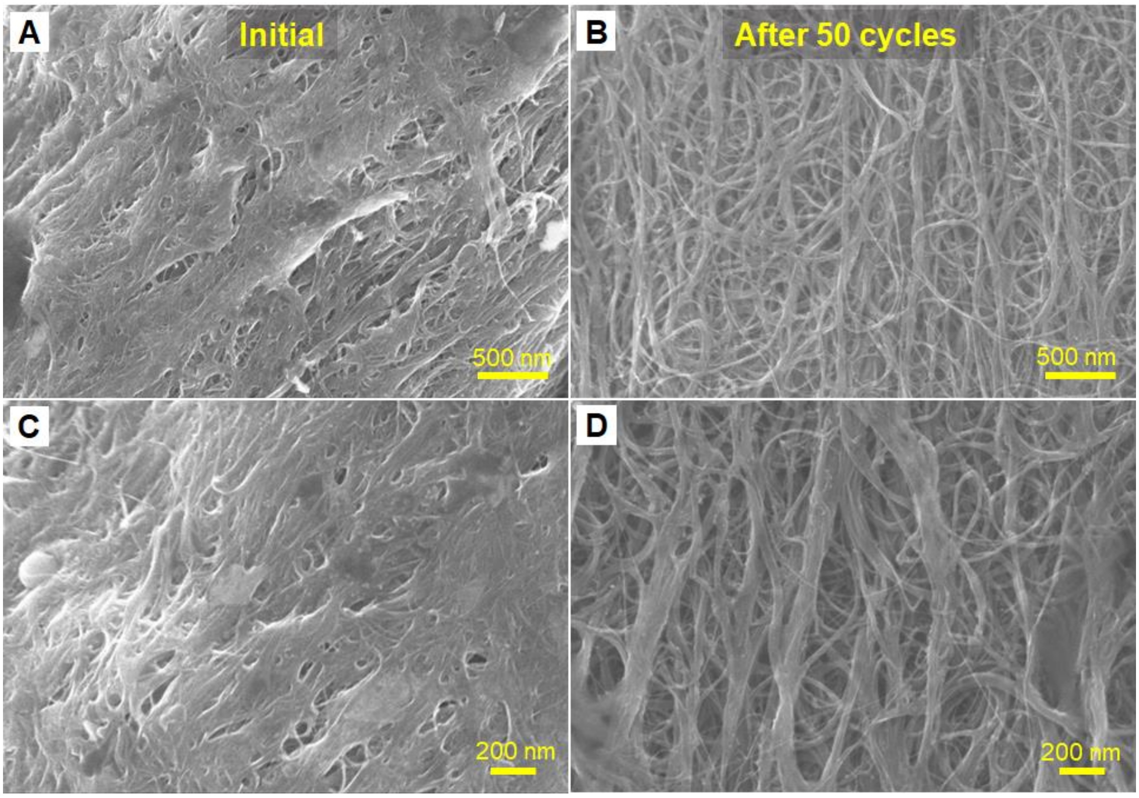

Fig. 1. Effect of mechanical twist/release cycling on the single-walled carbon nanotube (SWCNT) rope. Scanning Electron Microscopy (SEM) micrographs of y-rope (TPU) as produced (**A** & **C**) and after 50 twist/release cycles (**B** & **D**) in the different magnification.

Cycling under load strongly reduced the slack that had been present in the as-produced ropes. After the initial few cycles, the elastic response of the rope under loading and unloading reached a steady state, with no further structural alterations occurring in the SWCNT rope.

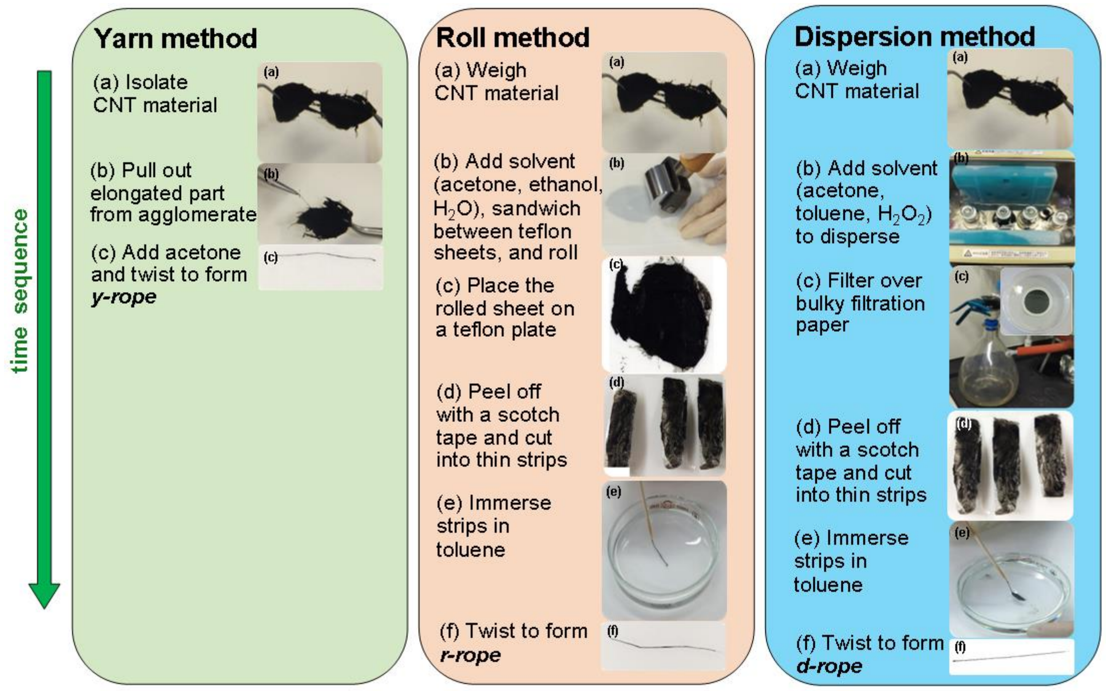

Fig. 2. Fabrication process of a SWCNT rope. Processing sequence for the yarn, roll and dispersion fabrication processes of SWCNT ropes.

Meijo eDIPS SWCNTs (EC2.0 and EC1.5, Nano Carbon Co. Ltd.) were employed as the primary material for the formation of SWCNT ropes.

The rope fabrication process via the yarn method involved extracting the longest SWCNT strand from the nanotube agglomerate using tweezers (a & b), followed by measurement of its weight and deposition onto a Teflon sheet. The application of a few drops of acetone onto each SWCNT strand led to their compaction. Subsequently, the compacted sample was manually twisted by several turns (c), resulting in the formation of what we term a **y-rope**.

For ropes produced via the roll process, a volume of less than 1 ml of the solvent (acetone, ethanol, or water) was applied onto 5-10 mg of the SWCNT material (a). The resulting agglomerate was then carefully positioned between Teflon sheets and compacted through rolling using a roller (b & c). A thin layer was subsequently extracted from the compacted SWCNT sheet using scotch tape (d). This thin layer was further divided into slender strips along the direction of the SWCNTs and submerged in toluene (e). The toluene-moistened strips were individually twisted by hand to create structures known as **r-rope** (f).

In the dispersion approach, approximately ≈1 mg of the material was suspended in 50 ml of the chosen solvent (acetone, toluene, or H_2O_2) through the process of sonication (a &

b). The resultant SWCNT dispersion underwent filtration and subsequent drying at 80°C to yield a bucky-paper structure (c). A delicate sheet of this bucky-paper was separated using scotch tape (d), then divided into strips and immersed in toluene (e). The toluene-saturated strips were individually subjected to manual twisting, giving rise to structures referred to as **d-rope** (f).

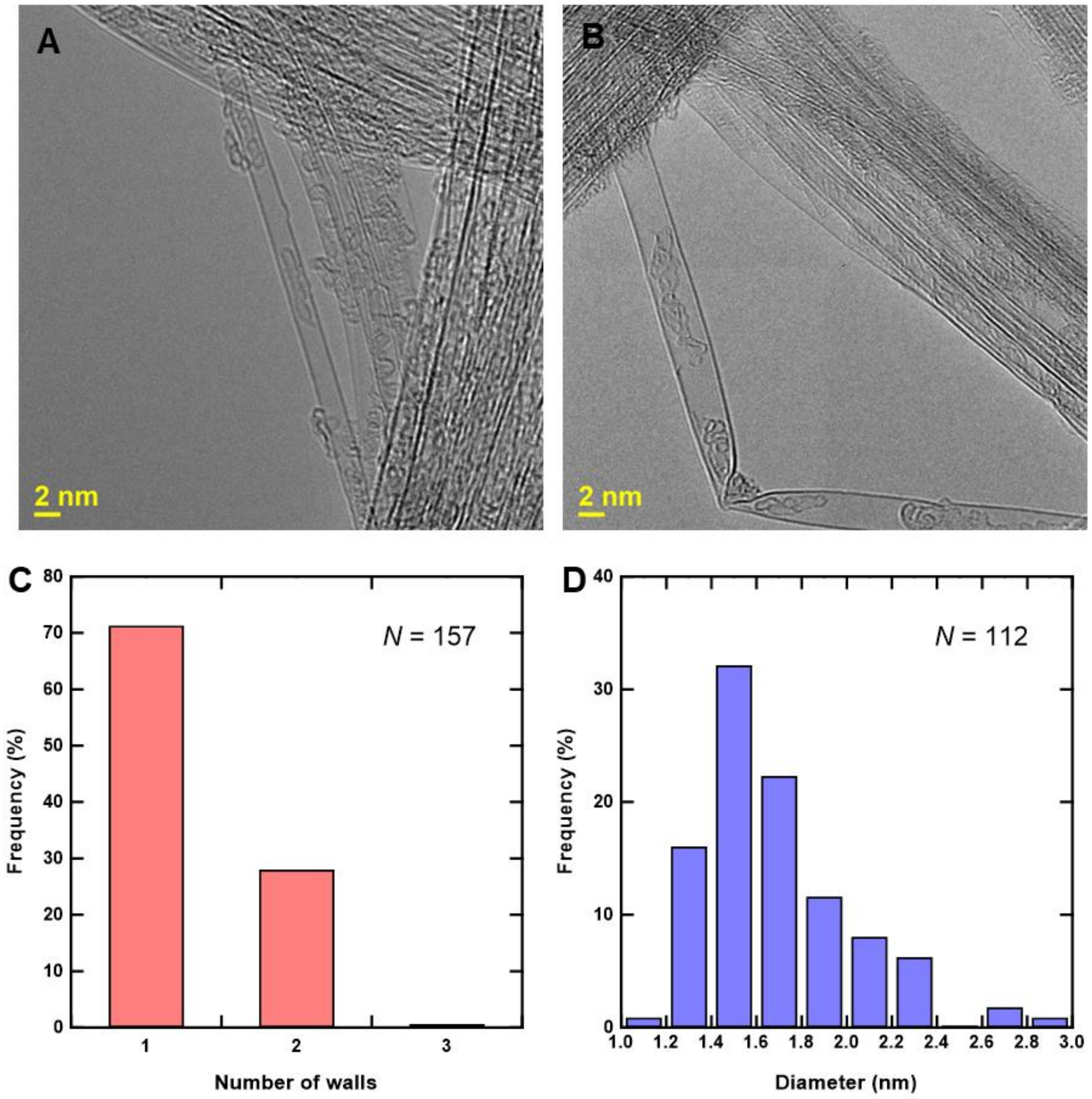

Fig. 3. SWCNT sample used to fabricate SWCNT ropes. (**A** & **B**) High-Resolution Transmission Electron Microscopy (HRTEM) micrographs of the starting material containing pristine SWCNTs (e-DIPS, EC1.5). (**C**) Statistical results for the number of walls of the starting CNTs and (**D**) for the diameter of SWCNTs.

The statistical results revealed an average tube diameter of 1.5 nm and predominantly single-wall configurations.

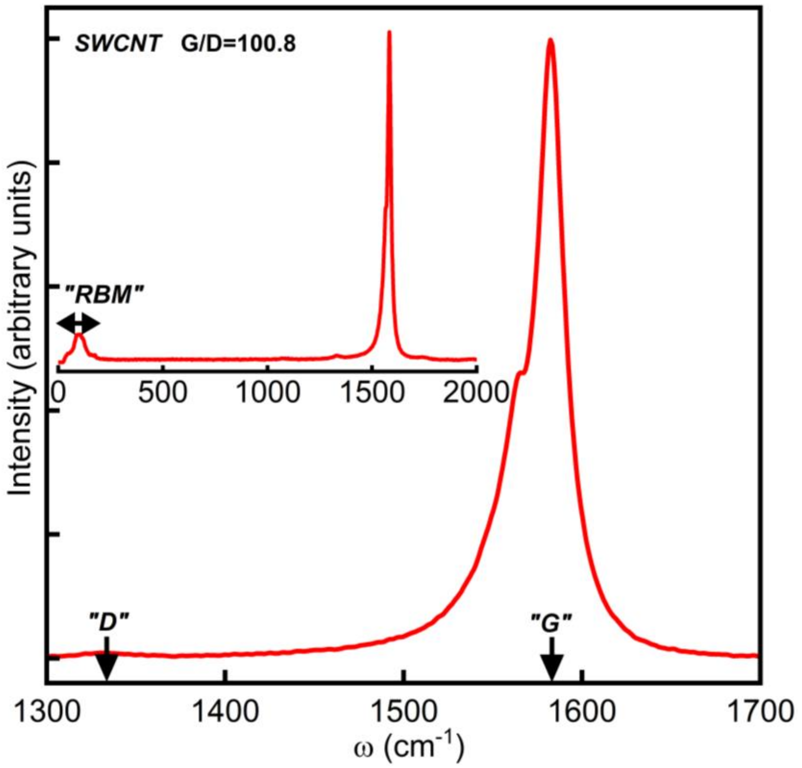

Fig. 4. Raman spectrum of pristine SWCNT. The Raman spectrum of the pristine SWCNT showing G/D ratio over 100. Raman spectroscopy measurement was performed using a 532 nm laser source.

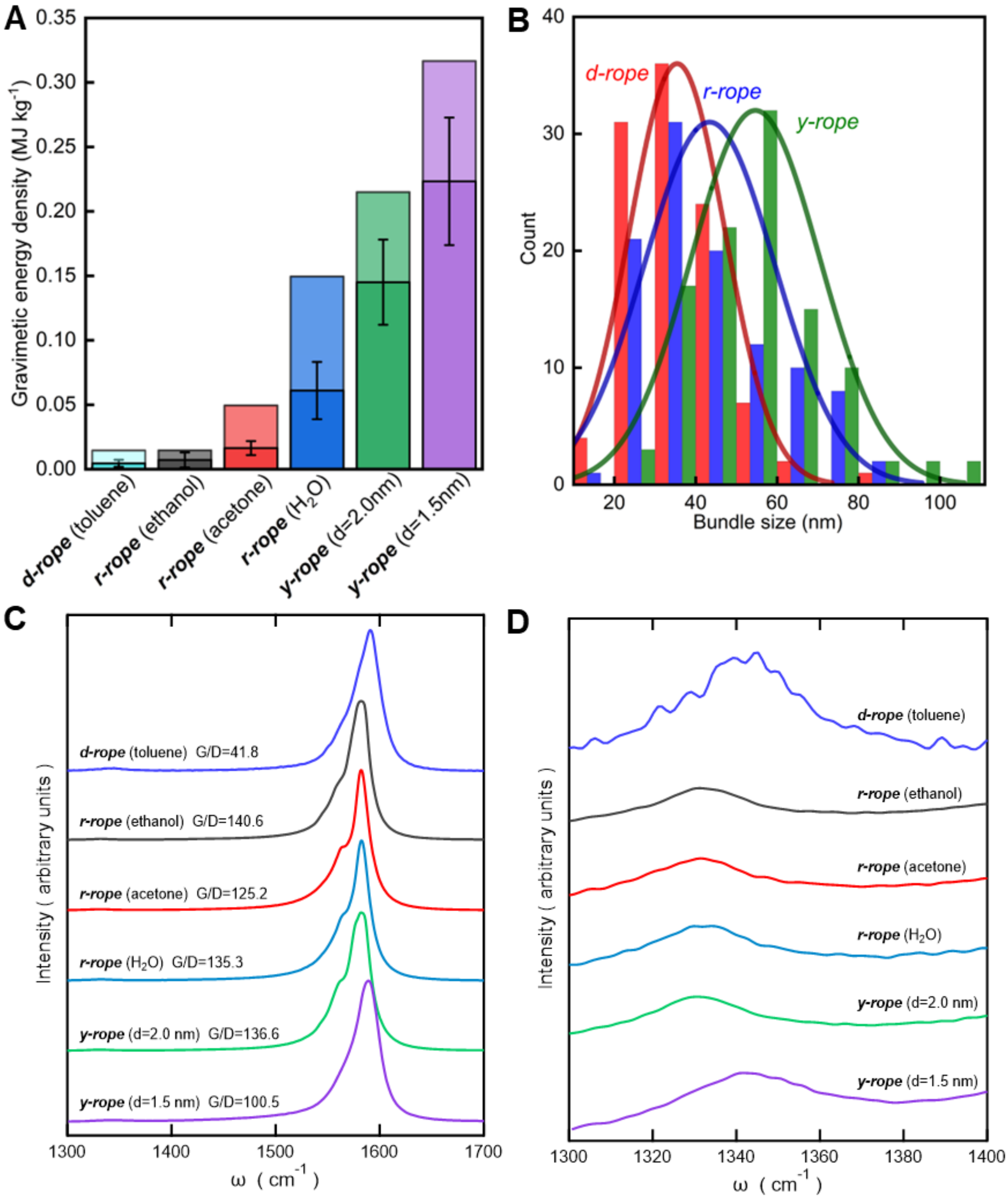

Fig. 5. Effect of processing method on the gravimetric energy density, tube bundling and the graphitic perfection of SWCNT ropes. (**A**) Comparative gravimetric energy density (GED) average (dark color bar) and maximum observed value (light color bar) in ropes fabricated using dispersion, roll and yarn methods. The data are presented as the mean ± standard deviation (s.d.) for $n = 5$ rope samples for each type. (**B**) The bundle size distribution determined using SEM micrographs of different types of SWCNT rope samples. (**C**) Raman spectra within the G- and D-band regions of the rope samples. Each spectrum is presented as a normalised relative value with respect to its G-band peak. (**D**) Magnified view of the D-band region.

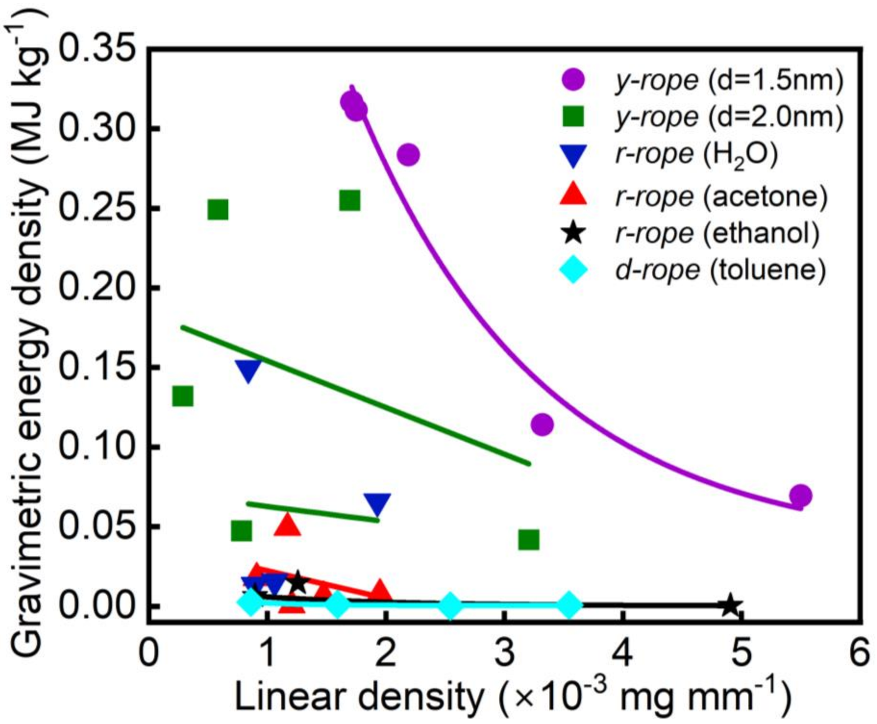

Fig. 6. Effect of processing method on the gravimetric energy density as a function of linear density of SWCNT ropes. Comparative gravimetric energy density (GED) of ropes fabricated using dispersion, roll and yarn methods with respect to their linear density.

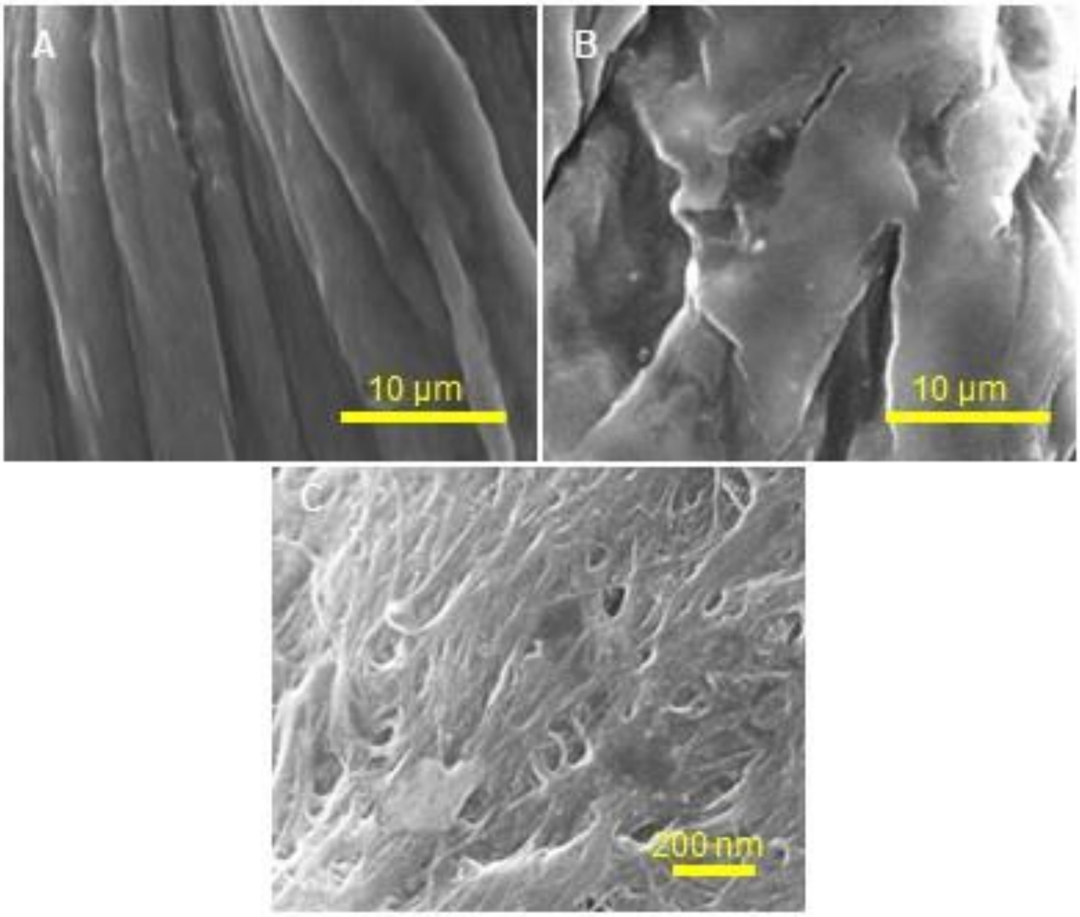

Fig. 7. Effect of microwave irradiation on the TPU-wrapped SWCNT rope. (**A**) SEM micrograph of TPU-wrapped y-rope before microwave irradiation. (**B**) Post microwave irradiation. (**C**) High magnification micrograph of microwave irradiated y-rope (TPU) showing polymer cover over the surface and interstitial sites of SWCNTs strands.

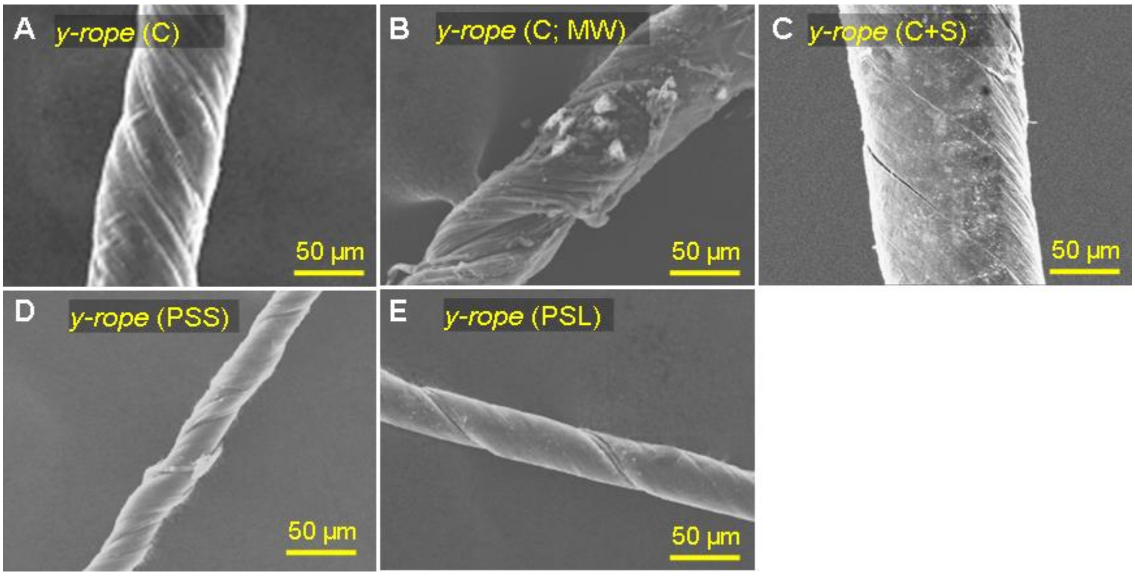

Fig. 8. Morphology changes in SWCNT y-ropes caused by modification using substances other than TPU. SEM micrographs of y-ropes exposed to carbon **(A)** prior to and **(B)** post microwave irradiation, **(C)** of ropes exposed to both carbon and sulfur, and of ropes exposed to **(D)** PSS and **(E)** PSL.

Polymers other than TPU we used to coat the ropes include short-polystyrene (PSS), with a molecular weight of 800-5,000 atomic mass unit (a.m.u.), and long-polystyrene (PSL), with a molecular weight of ≈300,000 a.m.u., both purchased from Polysciences.Inc. y-ropes exposed to carbon or sulfur showed a uniform level of carbon deposition across the surface, with no significant amount of carbon in the inter-yarn space.

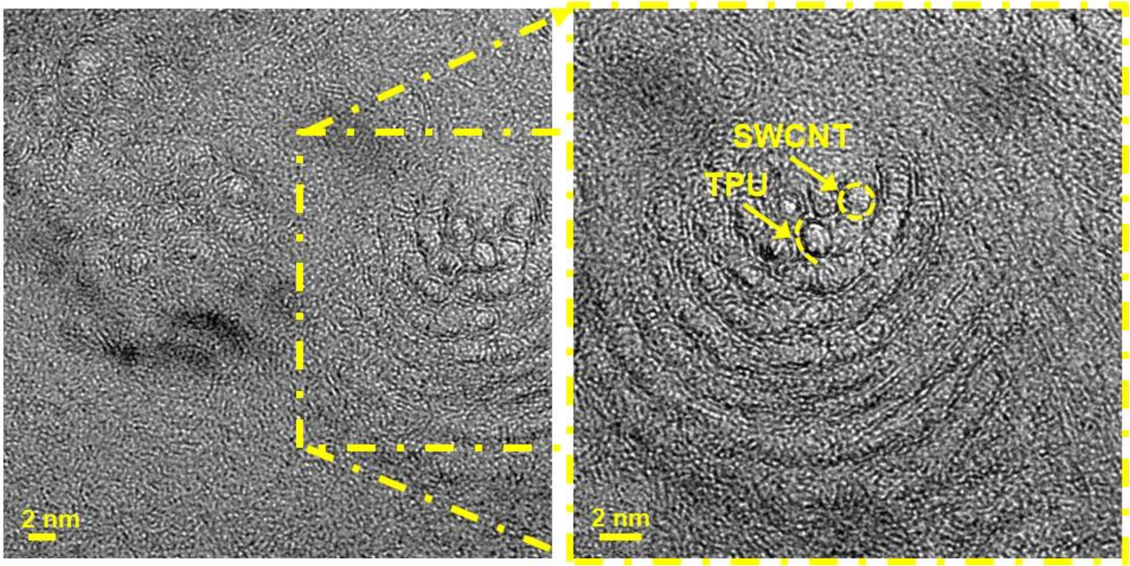

Fig. 9. HRTEM micrographs of y-rope (TPU). Cross-sectional HRTEM micrograph of the fabricated y-rope (TPU), indicating presence of SWCNTs uniformly wrapped with polymer layers. The yellow dashed circle and arc show the SWCNT and TPU cladding over the individual SWCNTs in the bundles. The uniform wrapping of the amorphous polymer layer on the surface and interstitial sites of the SWCNTs (lighter colour than the SWCNT sidewall) can be further observed. Similar morphological changes were observed for the other thermoplastic polymer-modified y-ropes.

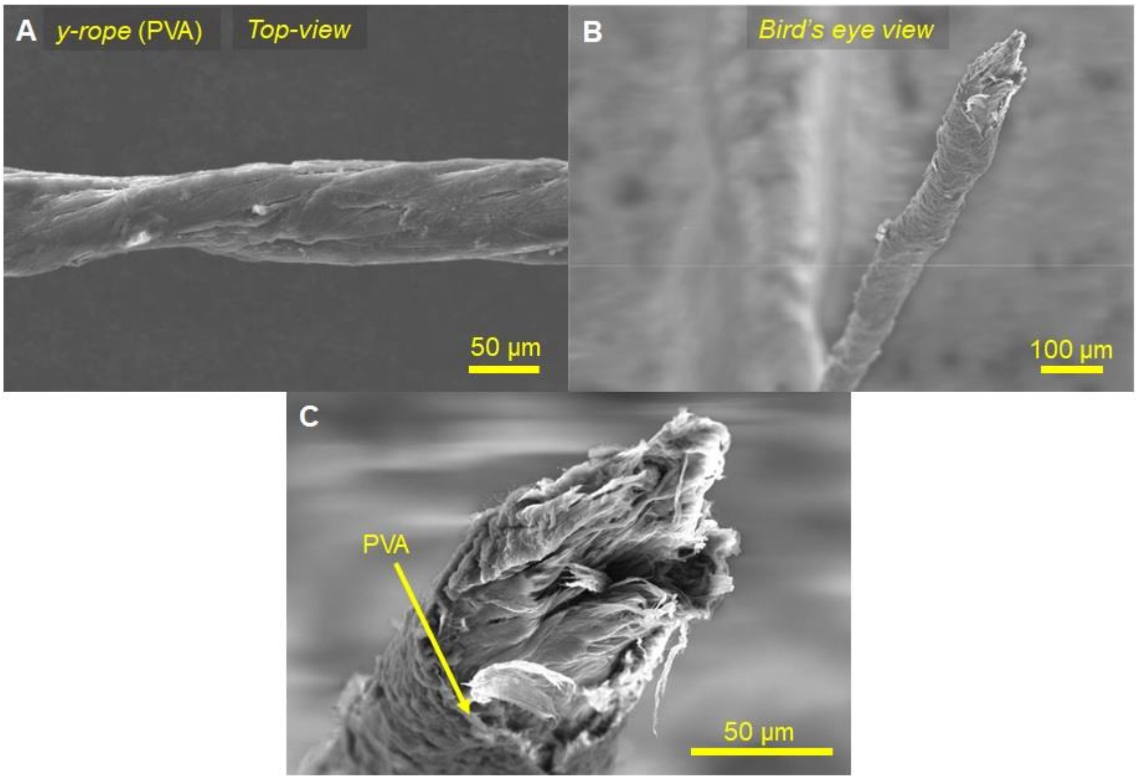

Fig. 10. SEM micrographs of y-rope (PVA). (**A**) Top view. (**B**). Bird's eye view of the fracture surface. (**C**). Magnified bird's eye view showing PVA cover over the SWCNT ropes like a co-axial structure.

Hydrophilic PVA does not interact strongly with hydrophobic SWCNT strand and they cannot soak between the SWCNT bundles. Then PVA forms own film covering the SWCNT strand. Then, PVA is not appropriate for improving the mechanical strength of the SWCNT ropes.

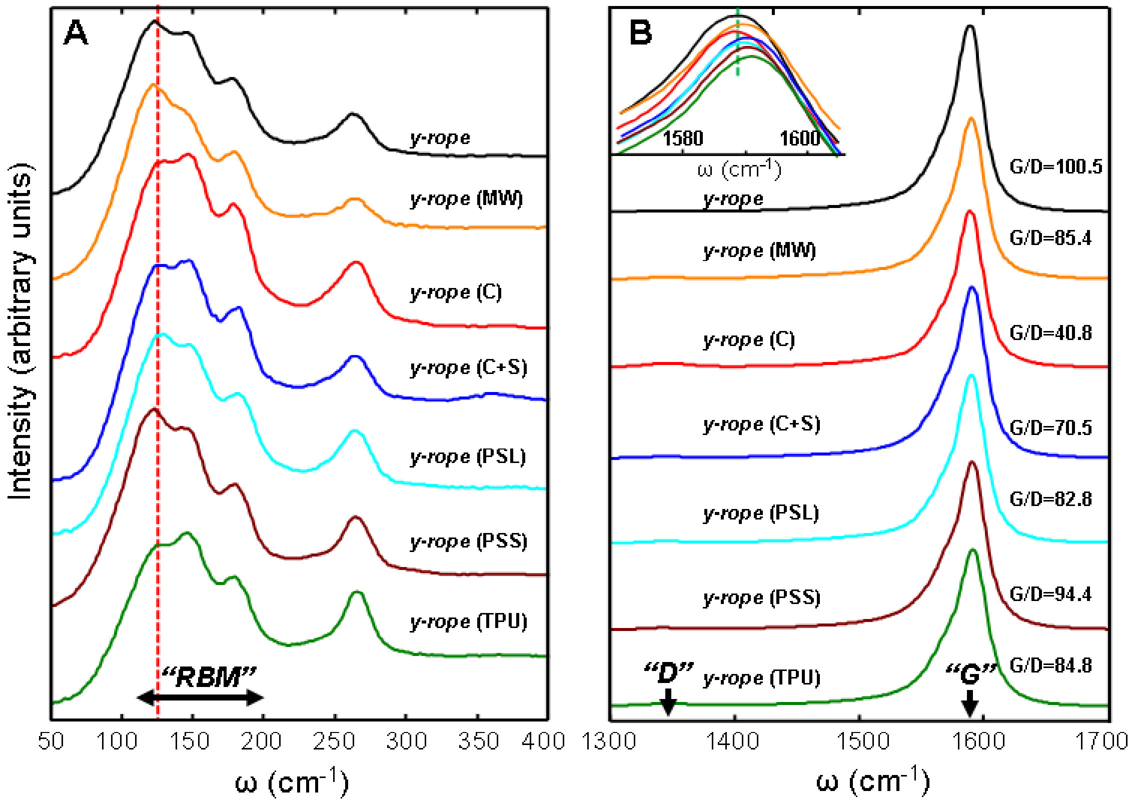

Fig. 11. Raman spectra of pristine and functionalized SWCNT ropes. **(A)** Raman signal in the frequency range associated with the radial breathing mode (RBM) of carbon nanotubes. The symmetry reduction in the rope causes a significant broadening of the radial breathing mode (RBM), which is well defined in individual SWCNTs, in the ω ≈ 100–300 cm-1 frequency range. **(B)** Raman signal in the frequency range associated with the "D" and "G" modes of graphitic structures. The inset illustrates the up-shift in the G-mode frequency induced by chemical functionalization. The pristine y-rope was well graphitised, with a high G/D ratio of approximately 100. The modification with TPU, PSS, and PSL lowered the G/D ratio to some degree. The deposition of carbon and sulfur on the ropes caused a significant decrease in the G/D ratio, indicating the formation of defective or amorphous structures, along with the deterioration of the mechanical properties of these structures.

Table 1. G-mode frequencies in pristine and modified SWCNT ropes

SWCNT rope system	Frequency ω
y-rope (pristine)	1589.0 cm^{-1}
y-rope (MW)	1590.1 cm^{-1}
y-rope (C)	1589.3 cm^{-1}
y-rope (C+S)	1591.0 cm^{-1}
y-rope (PSL)	1590.8 cm^{-1}
y-rope (PSS)	1590.9 cm^{-1}
y-rope (TPU)	1591.7 cm^{-1}

Observed Raman spectra in Fig. 11A indicate significant broadening of the RBM due to symmetry reduction in the rope. Signal intensity ratio in the range of the G (graphitic) and D (disorder) modes, indicated in Fig. 11B, is used to judge the graphitization of the material, with high G/D ratios indicating high quality. Chemical functionalization improves alignment and increases packing within the rope, evidenced in the up-shift of the G-mode frequency.

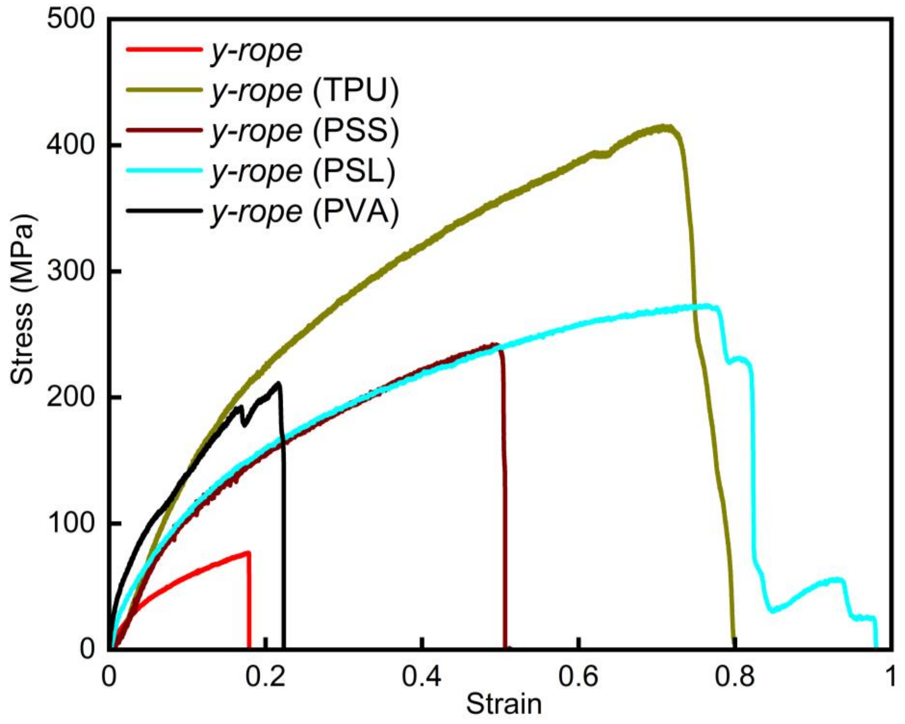

Fig. 12. Mechanical properties of y-rope samples. Stress-strain curves of y-rope and various polymer modified y-rope samples.

Table 2. The mechanical properties of the y-rope and polymer modified y-ropes, evaluated from their stress vs strain curves. The data are presented as the mean ± s.d. for $n = 3$ rope samples for each type.

Rope Sample	Young's modulus (GPa), E	Tensile strength =Breaking strength (MPa), σ_B	Elongation, ε_B
**y-rope**	1.6±0.54	80±11	0.13±0.06
**y-rope (TPU)**	2.1±0.26	420±31	0.60±0.21
**y-rope (PSS)**	1.9±0.50	250±26	0.35±0.22
**y-rope (PSL)**	2.9±0.87	390±51	0.54±0.25
**y-rope (PVA)**	3.0±0.65	400±140	0.22±0.09

Hydrophilic PVA modification improved mechanical properties but not as much as the other polymers employed. This phenomenon may be attributed to the presence of PVA primarily on the hydrophobic surface of the y-rope, whereas thermoplastic polymers with hydrophobic characteristics diffuse into the inter-bundle spaces, interconnecting the SWCNTs and enhancing their load-transfer capabilities in the modified y-ropes. This, in turn, induces elastic distortion in the individual SWCNTs, resulting in high mechanical energy storage.

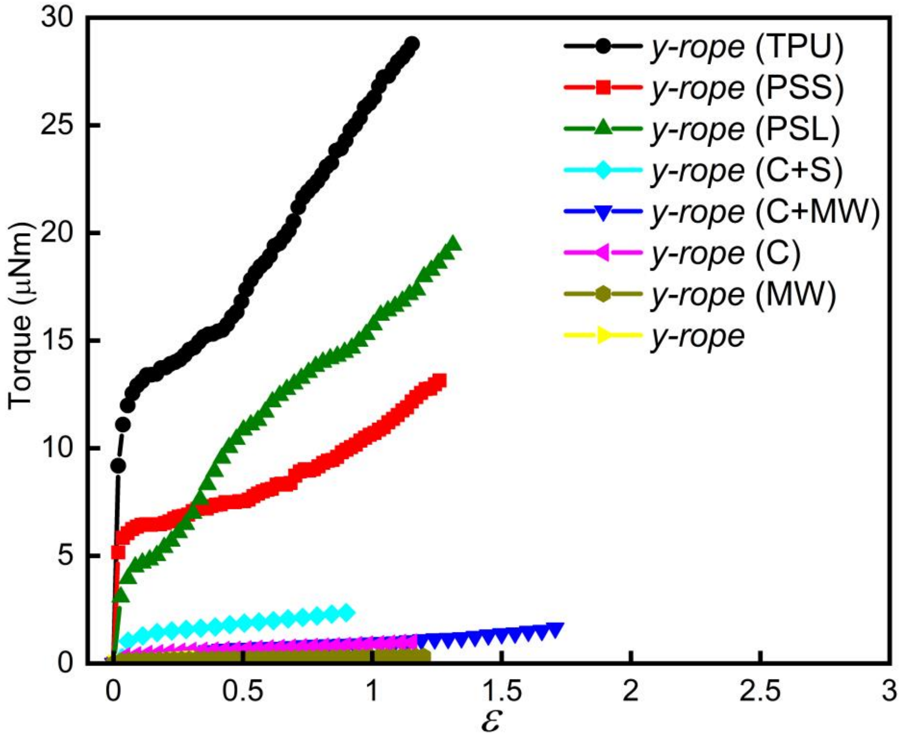

Fig. 13. Torque as a function of the torsional strain ε. Torque generated in y-rope and modified y-rope samples of comparable diameter 30±4 μm during torsional strain.

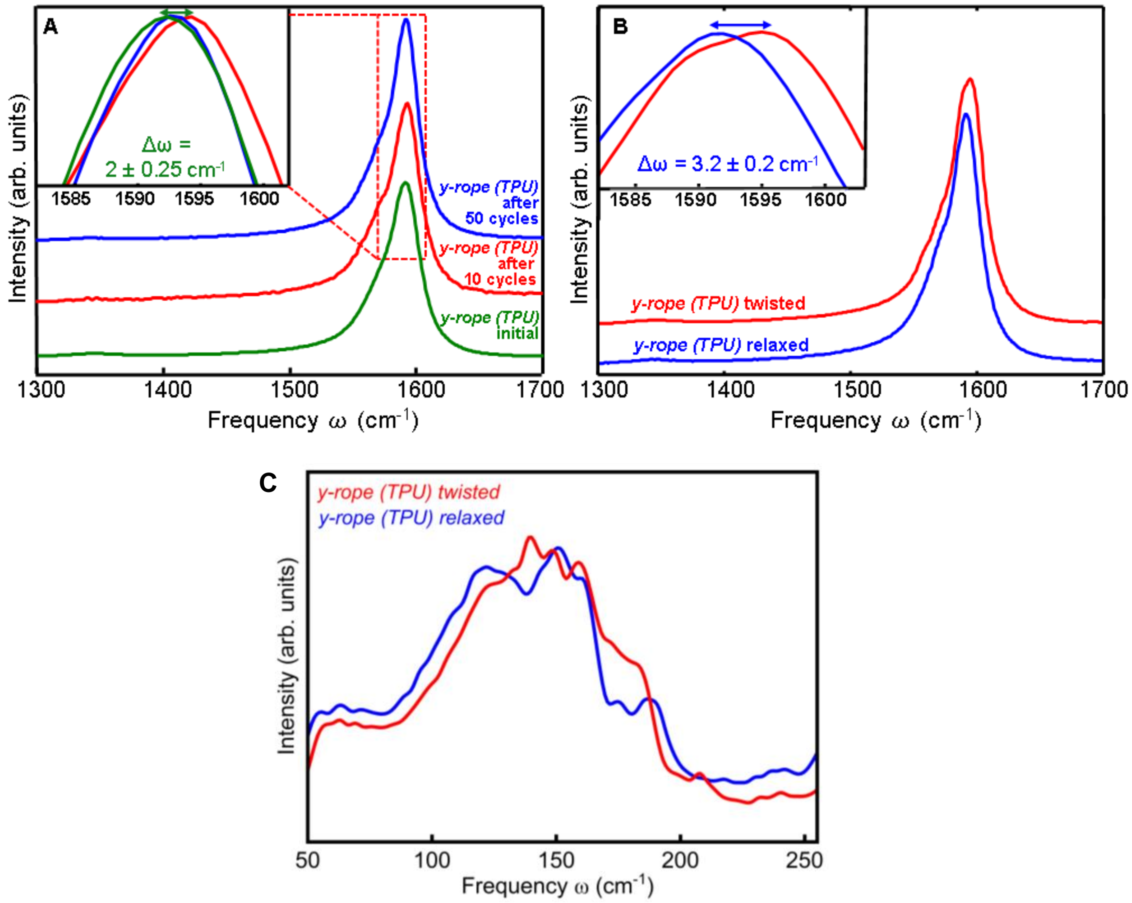

Fig. 14. Structural change during mechanical twist/release cycling. In-situ Raman spectra of a y-rope (TPU). (**A**) Spectra of a rope that has been subjected to different numbers of twist/release cycles. Details of the frequency shifts in the G-peak area are shown in the inset. (**B**) Reversible up-shift observed in the G-mode frequency of a y-rope (TPU) caused by twist. (**C**) Shift observed in the frequency range associated with the radial breathing mode (RBM) of a y-rope (TPU) during twisting.

The observed shift in the G-peak can be attributed to intertube load transfer and tube structure distortion, working in conjunction rather than exclusively. The relationship between the G-peak position and the radial breathing mode (RBM) peaks in the context of intertube load transfer and tube structure distortion can provide further insights into the conclusion. Although they do not directly distinguish between these two phenomena, their combined analysis can offer insights into the underlying mechanisms. The G-peak position is primarily influenced by the in-plane vibrations of the sp^2 carbon atoms in the CNT lattice. Changes in the G-peak position can occur due to several factors, including strain, doping, defects, and intertube interactions. In the case of intertube load transfer

and distortion to the tube structure, the RBM-peaks, which arise from the radial vibrations of the CNTs, can provide additional information. If the G-peak shift is primarily a result of intertube load transfer, it is expected that the RBM-peaks will exhibit similar changes. This is because intertube load transfer affects the overall strain and distortion in the CNT structure, which will influence the radial vibrations and, consequently, the RBM-peaks. However, if the RBM-peaks remain relatively unchanged while the G-peak shifts, it supports the hypothesis that the G-peak shift is predominantly driven by tube structure distortion rather than the intertube load transfer. Fig. 14B&C, which displays the shift of the RBM- and G-peaks under relaxed and twisted states, provides clear evidence of the significant changes in the RBM-peaks concurrent with the shift in the G-peak. This observation strongly suggests the occurrence of intertube load transfer within the structure of the SWCNT ropes.

In a rope under tension, initially misaligned nanotubes are pressed against each other, with the maximum pressure occurring at the crossing points where the G-modes locally harden, shifting the G-band to a higher average frequency. As the nanotubes were gradually aligned during additional twist/release cycles, the crossing points were eliminated, resulting in a G-band upshift. In fact, the G-band frequency recovered its initial value after approximately 50 cycles and remained constant. This suggests that there are no structural changes or long-term stability when y-rope (TPU) is used in practical energy storage applications.

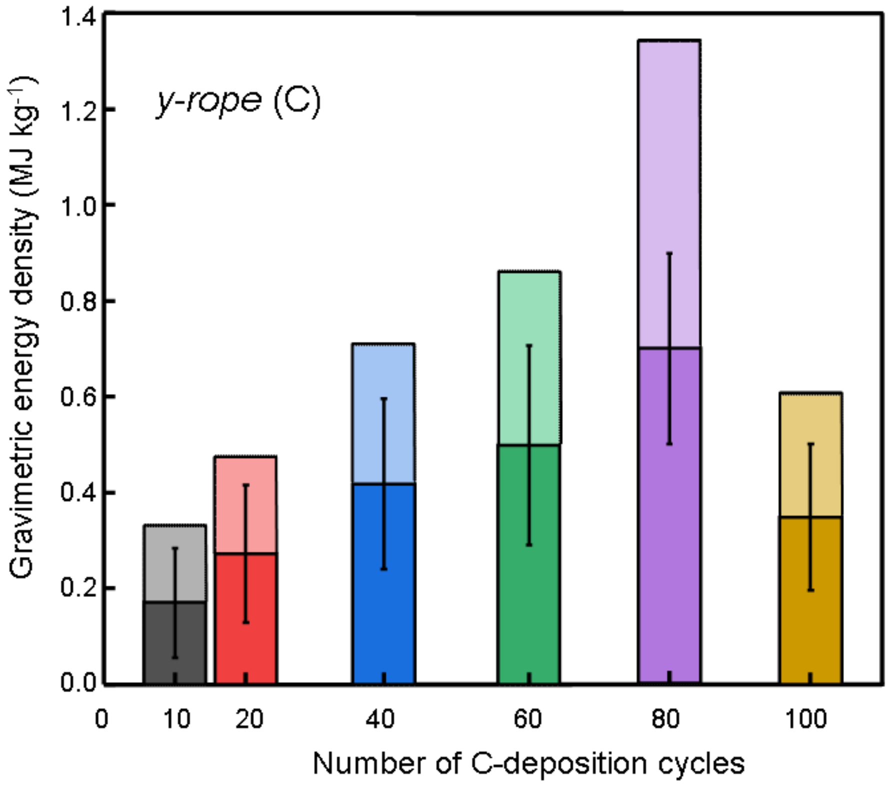

Fig. 15. Effect of carbon deposition on the maximum GED of SWCNT y-ropes (C). GED of y-rope (C) as a function of the number of carbon deposition cycles. Average GED values are represented by dark color bars and maximum values by light color bars. The data are presented as the mean ± s.d. for $n = 5$ y-rope (C) samples for each type.

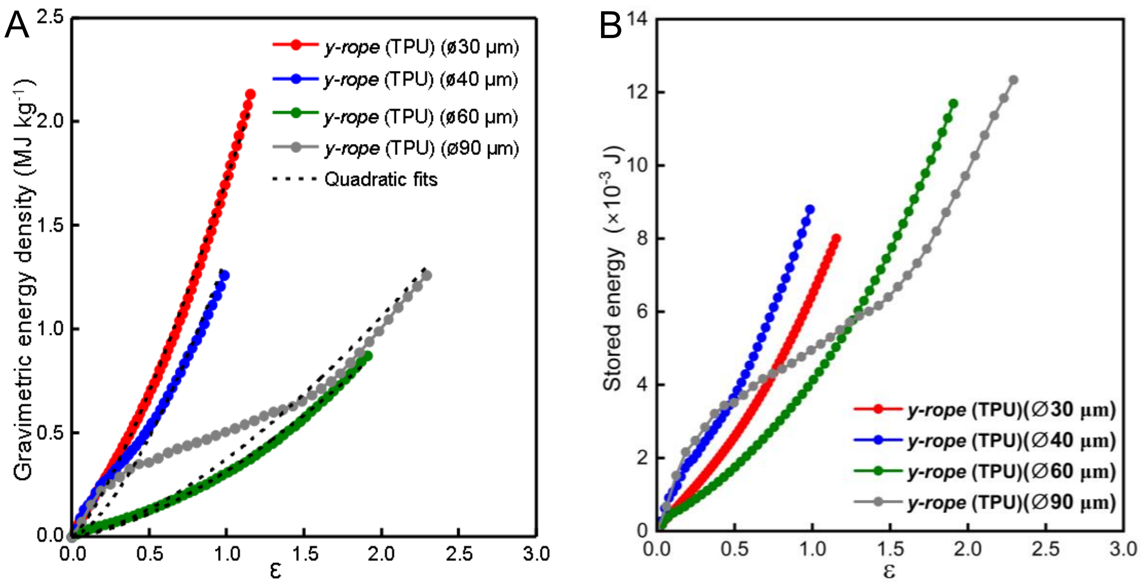

Fig. 16. GED [MJ/kg] (A) and total stored energy [J] (B) in y-ropes (TPU) of different diameter. GED in y-ropes (TPU) with cross-sectional diameters ranging from 30–90 μm as a function of the torsional strain ε. Quadratic fits to the data, shown by the dotted lines, illustrate agreement with Hooke's law for torsional springs.

The GED – ε relationship shown in Fig. 16A indicates that highest energy densities are expected in narrow ropes. The strongly anharmonic behavior in the widest rope with a diameter of 90 μm is indicative of irreversible plastic deformations at high torsional load, as shown in Fig. 17.

Nanomechanical energy reversibly stored in twisted SWCNT ropes consist of stretching, twisting, bending, and compression, each with a different elastic limit. The elastic regime is limited by the twisting of individual SWCNTs in narrow ropes and the stretching of the outermost SWCNTs in wide ropes. With increasing rope diameter, we find a decrease from k/m = 1.9±0.0099 MJ kg^{-1} in 30-μm ropes to 0.27±0.013 MJ kg^{-1} in 90-μm ropes.

In this context, it is pertinent to discuss the actual cumulative energy storage (in J) rather than the GED, considering the practical application potential of SWCNT ropes. The cumulative energies stored within y-ropes (TPU) of varying diameters corresponding to the GED values presented in Supplementary Fig. 16A are shown in Fig. 16B. Despite their relatively modest magnitudes owing to the modest size of the rope, the cumulative energy increased proportionally to the increase in the rope diameter. This phenomenon can be attributed to the fact that twisting a thicker rope generates a higher torque. This observation augments the practical viability of SWCNT ropes, as it increases the rope diameter, and employing multiple ropes in parallel, inevitably elevates the cumulative stored energy. A thicker rope has a larger cumulative stored energy, as shown in Fig. 16A,

indicates an increase of over twofold (30×10^{-3} J). However, it is noteworthy that thicker ropes hindered the load transfer within the SWCNT structure, thereby posing a GED-related drawback.

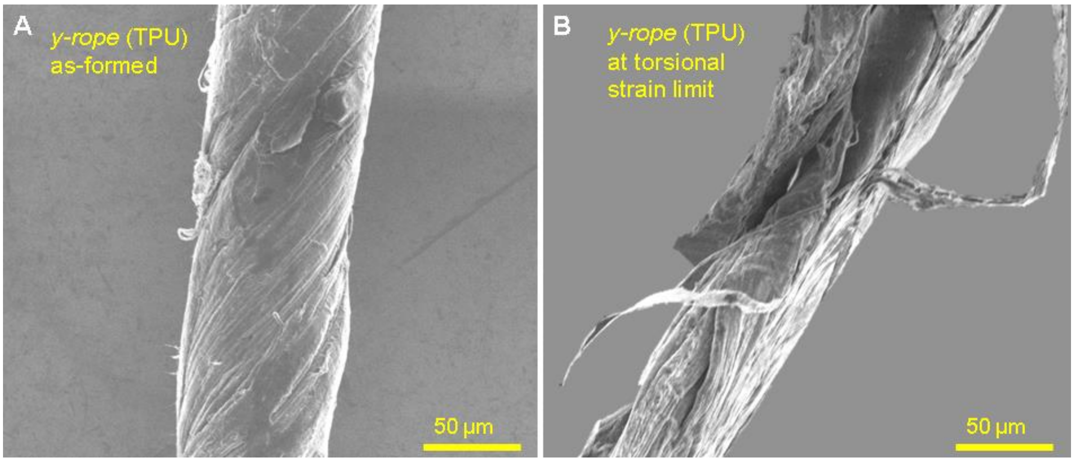

Fig. 17. Morphology change in a y-rope (TPU) at the torsional strain limit. SEM micrographs of a TPU-modified y-rope with a diameter of 90 µm (**A**) in the initial state and **(B)** at the torsional strain limit. Rupture is initiated in the outermost SWCNT bundles at the rope surface.

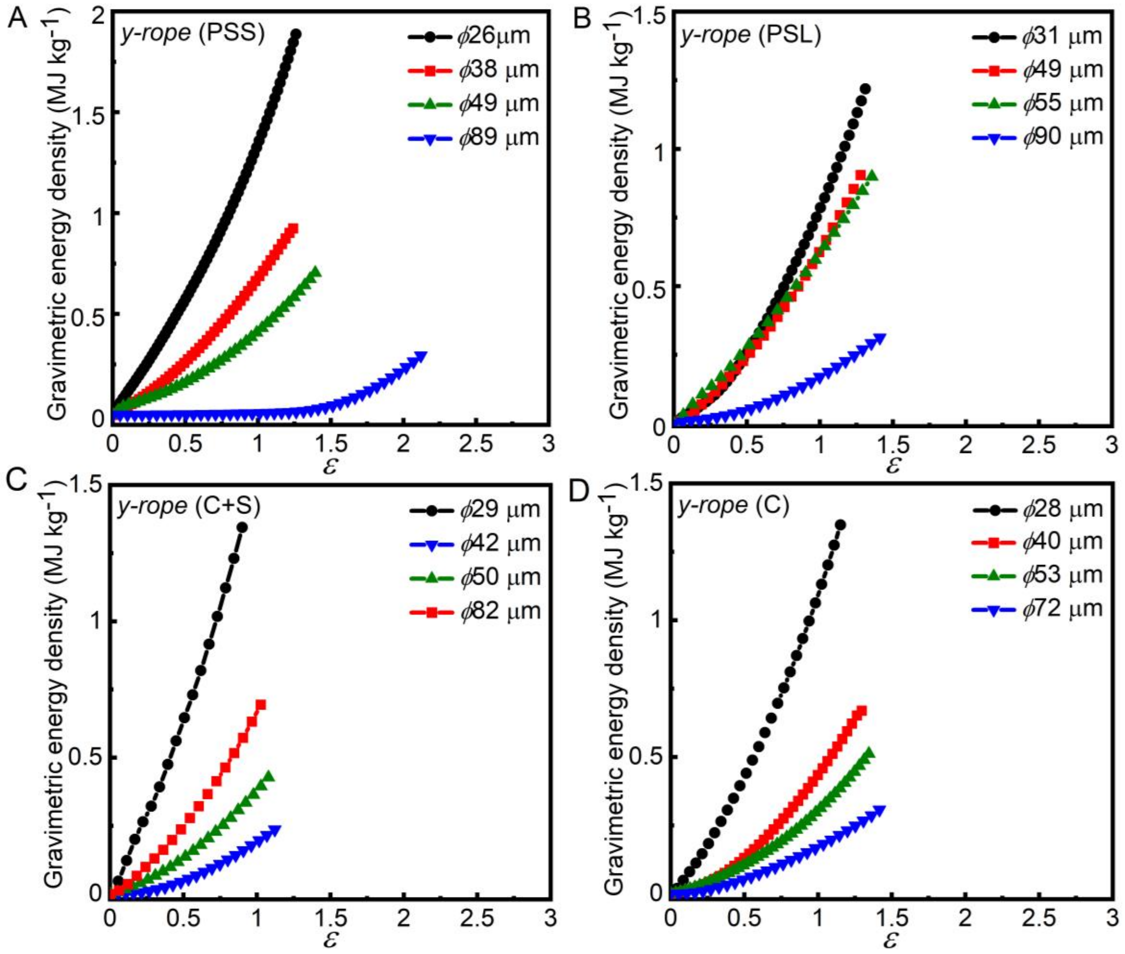

Fig. 18. GED in modified y-ropes of different diameter. GED in (**A**) y-rope (PSS), (**B**) y-ropes (PSL), (**C**) y-ropes (C+S) and (**D**) y-ropes (C) with various cross-sectional diameters as a function of the torsional strain ε.

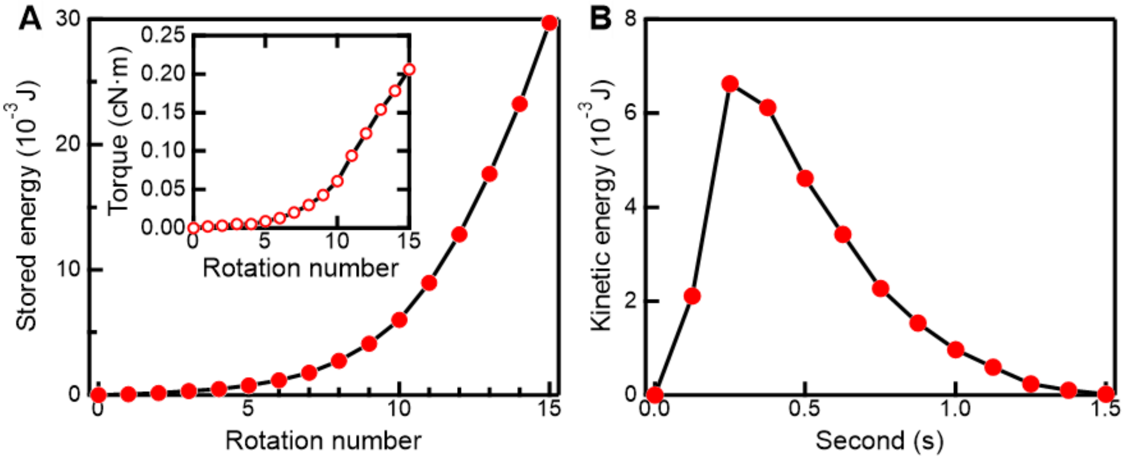

Fig. 19. Energy conversion efficiency for circular disc rotation. (**A**) Energy stored in ten y-ropes (TPU) samples twisted together for 15 rotation cycles. Inset shows the increase in torque with increasing rotation numbers. (**B**) Kinetic energy of the circular disc utilizing the mechanical energy stored in the twisted rope samples during 15 rotation cycles.

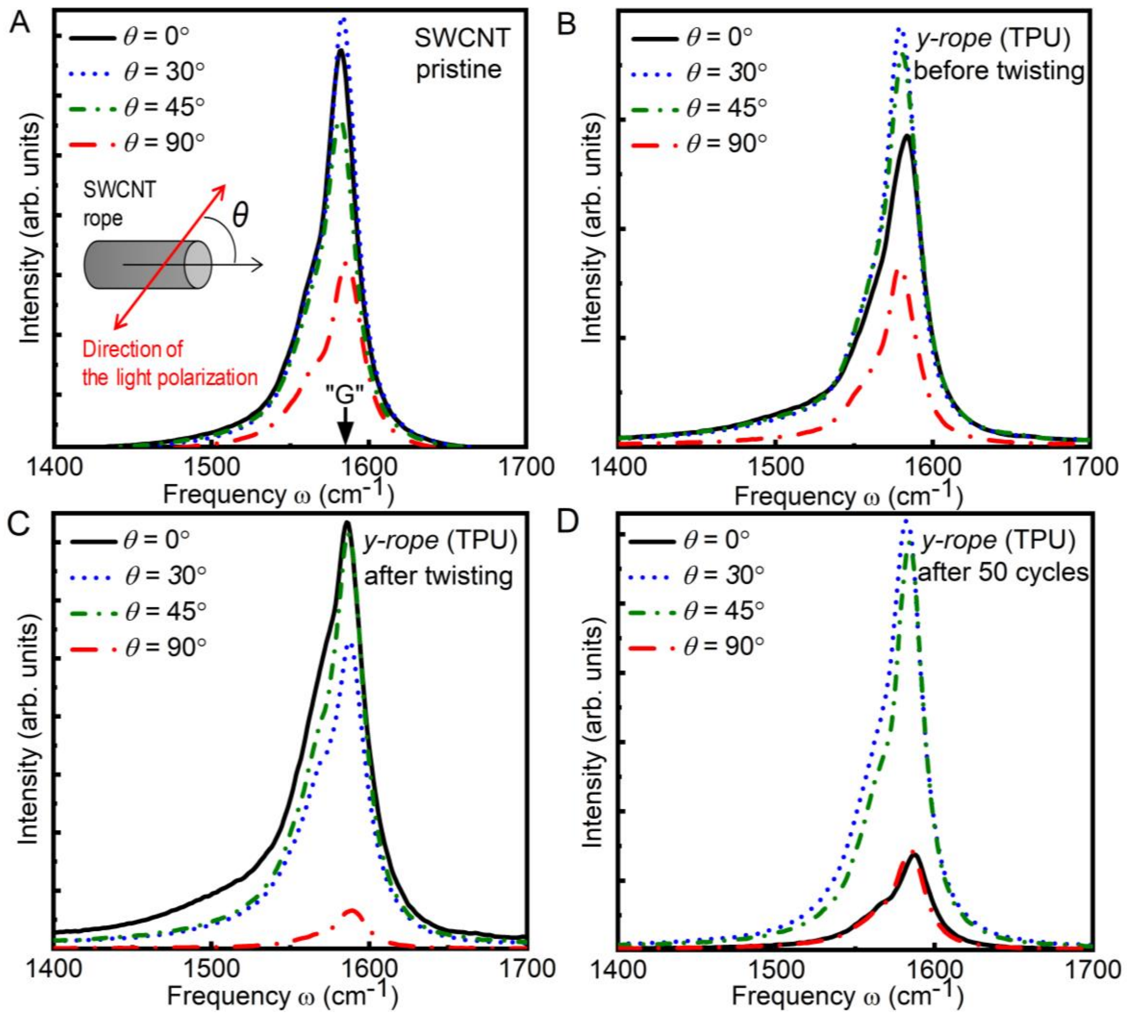

Fig. 20. Polarized Raman spectra of pristine SWCNT and y-rope (TPU) with relevance to the alignment of SWCNT. (**A**) Raman spectra "G" band of pristine SWCNT at angles θ = 0°, 30°, 45° and 90°. Inset showing the schematic illustration of the angle between the polarization direction and the SWCNT rope axis. (**B**) Raman spectra "G" band of y-rope (TPU) before twisting at angles θ = 0°, 30°, 45° and 90°. (**C**) Raman spectra "G" band of y-rope (TPU) after twisting at angles θ = 0°, 30°, 45° and 90°. (**D**) Raman spectra "G" band of y-rope (TPU) after 50 twist/release cycles at angles θ = 0°, 30°, 45° and 90°.

Here, we measured the polarization effect at 4 points in the rope. Figures above show dependence of the "G" band intensities vs. the polarization angles, indicating that the pristine SWCNT do not have completely uniform alignment. The angle dependence of "G" band intensity on TPU coating remained similar to that of the pristine SWCNT, signifying that the coating enables to embrace short SWCNT bundles to a long rope, but do not enhance local alignments. However, twisting the TPU-coated y-rope induces

slightly the alignment of the SWCNTs in the y-rope (TPU). The “G” bands intensities between at $\theta = 0°$ and 45° are highest, whereas at $\theta = 90°$ is quite low; signifying that SWCNTs are mostly aligned along the lower angles. This alignment effect gets even more prominent and narrow centered at $\theta = 37°$, after 50 repeated twist/release cycles. During these preconditioning cycles SWCNTs aligned themselves due to relaxation effect to attain most stable configuration along $\theta = 30°$ and 45°. This suggests that first twisting to develop rope configuration and the repeated preconditioning twist/release cycling enhances the local alignment of the SWCNTs and bring them in their most relaxed state; to store energy stably hereafter.

The normalised “G” band intensity as a function of “θ” (the angle between the light of polarisation direction and the long axis of the SWCNT) show clear distinction between the pristine SWCNT and rope samples. The “G” band intensities of pristine SWCNT at angles $\theta = 0°$, 30°, 45° and 90° showed no significant differences, indicating non-uniform alignment of the SWCNTs.

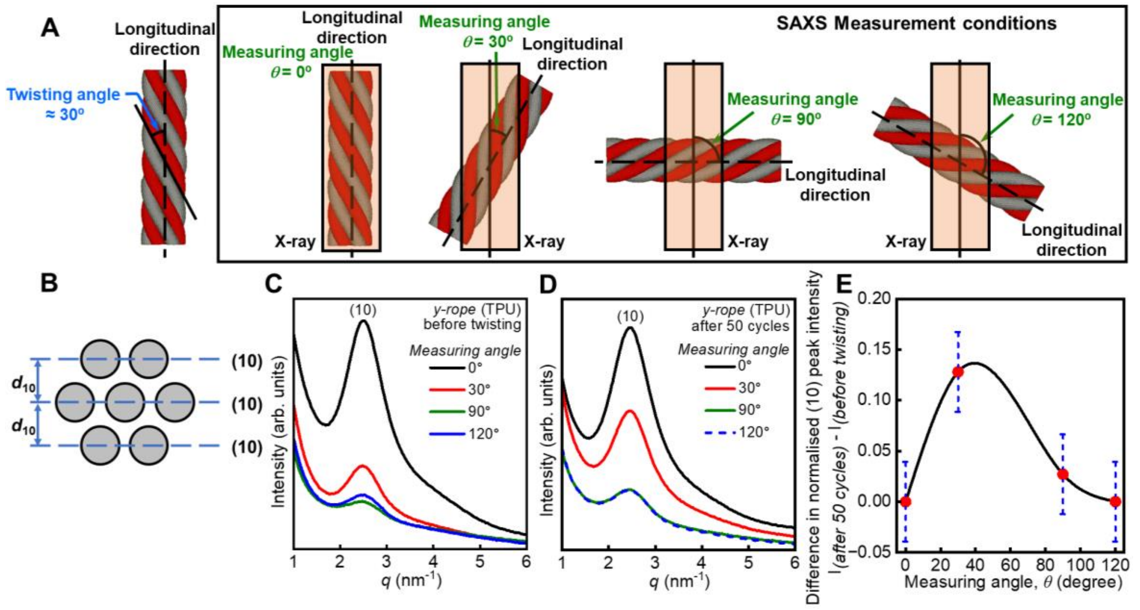

Fig. 21. Synchrotron small-angle X-ray scattering (SAXS) measurement of y-rope (TPU) samples with relevance to the alignment of SWCNT bundle. (**A**) Schematic representation of the measuring angle (θ) between the X-rays beam and the longitudinal direction of the twisted y-rope (TPU) samples. (**B**) Interplanar spacing for the peak at 2.51 nm^{-1} corresponding to the reflection due to the (10) planes of the SWCNT bundle structure. (**C**) The SAXS diffractograms of y-rope (TPU) at measuring angles θ = 0°, 30°, 90° and 120°, before twisting and (**D**) after 50 twist/release cycles. The peak intensity of the (10) reflection of SWCNT bundles of the y-rope (TPU) at the measuring angle of 0° is the highest, because the largest area of the SWCNT rope is exposed to X-rays. (**E**) The measuring angle dependence of the (10) peak intensity-difference between after 50 twist/release cycles and before twisting for the y-rope (TPU). Here, the peak intensities are normalised by dividing the intensity at each measuring angle by the intensity at 0° after calibration of the X-ray-irradiated area difference. The peak intensity-difference was then obtained by subtracting the intensity before twisting ($I_{\text{before twisting}}$) from the intensity after 50 cycles twisting ($I_{\text{after twisting}}$). The data are presented as the mean ± s.d. for n = 3 y-rope (TPU) samples.

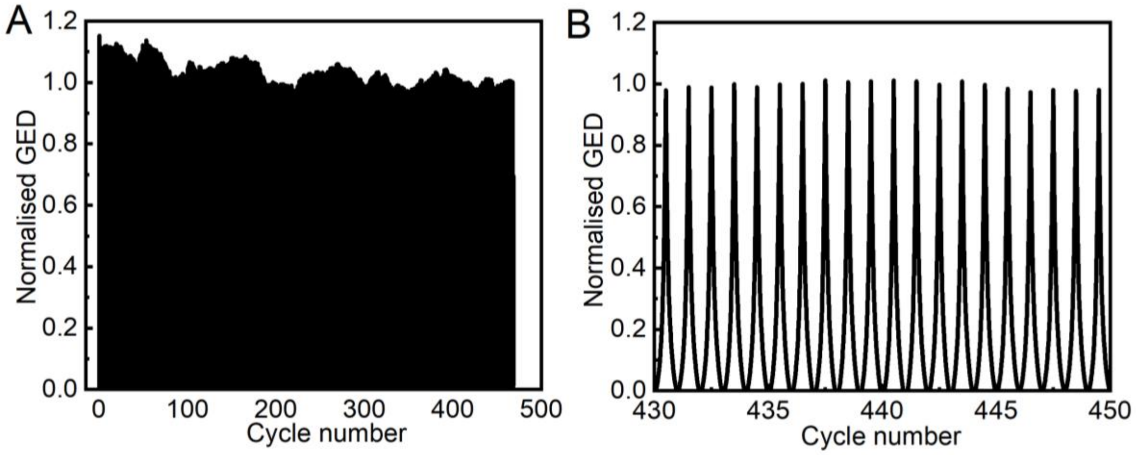

Fig. 22. Cycling stability of the y-rope (TPU) during over 450 consecutive twist/release cycles. (**A**) The normalised GED is measured up to a maximum torsional strain ε = 0.6 at a rotational frequency of 110 rotation per minute (rpm). The measurements are performed after initial preconditioning twist/release. (**B**) Magnified view demonstrating few cycles exhibiting consistently maintain GED over 450 twist/release cycles, underscoring their remarkable stability.

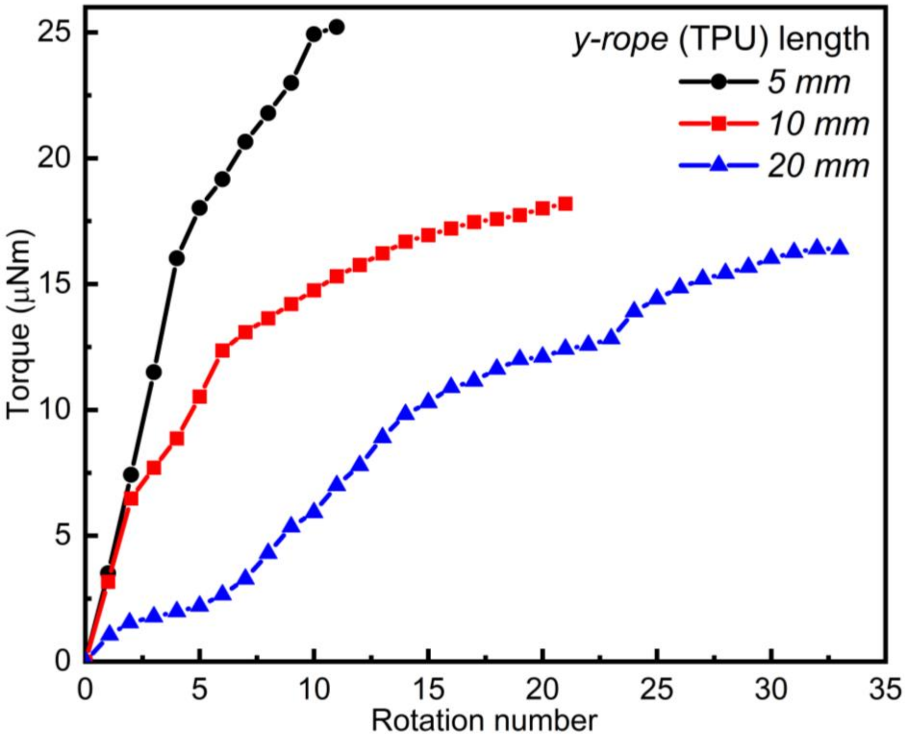

Fig. 23. Measurement length dependence of SWCNT rope on torque. Torque as a function of the rotation number for y-rope (TPU) samples of the length (eye hook to eye hook) of 5 mm to 20 mm.

Experiments are performed for understanding the relationship between the rope length and torque using y-rope (TPU) having eye hook to eye hook length 5 mm, 10 mm and 20 mm. With increasing rotation, the torque and hence the resulting GED decreased. This is associated with the macroscopic defects in the SWCNT ropes, which deteriorated the mechanical properties of resulting rope samples. In order to confirm this, similar experiments were performed on carbon fibers (having minimum defect structure) ropes. The preliminary experiment indicated insensitive dependence of the torque against fiber length. The torques measured per unit length are almost similar. However, the most striking difference here is the magnitude of GED generated in the y-rope (TPU) and carbon fiber ropes. Under comparable conditions, the y-rope (TPU) generated a GED 10 times higher compared to the carbon fiber ropes. It signifies the importance of using SWCNTs for rope preparation; SWCNT based ropes can store GED very high compared to carbon fiber ropes. In future, we must improve the procedure to produce a long and uniform SWCNT based rope for generating larger GED for a long length of the rope sample.

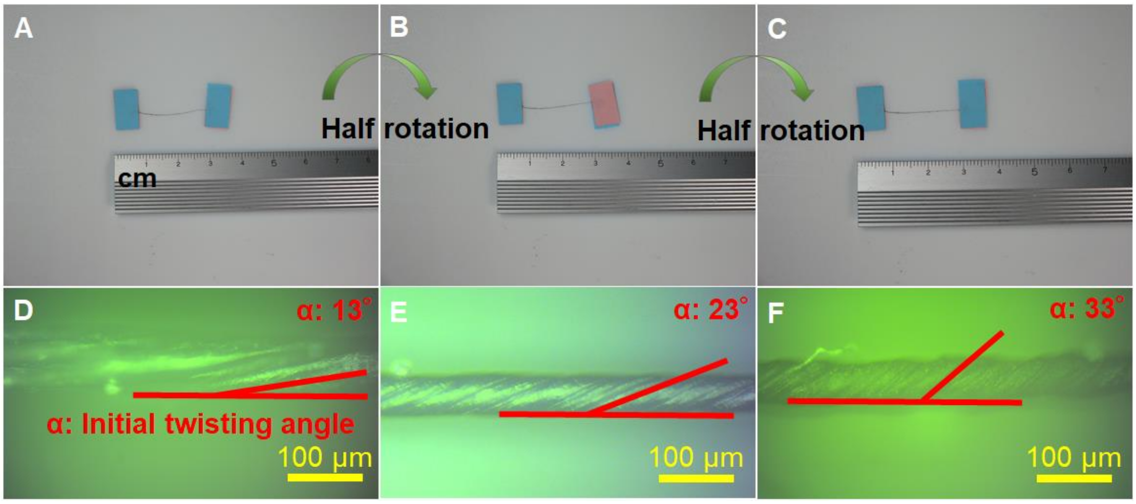

Fig. 24. **Twist angle determination during rope sample fabrication**. (**A-C**). Demonstrating twist rotations during the rope fabrication process. (**D-F**). Twist angles of the rope samples with increasing number of twist rotations.

The paper paddles are attached on both the ends of the y-ropes. One end is fixed and another end is twisted. After every few twists, the rope samples are observed under an optical microscope to determine the twist angle (α) with increasing twisting numbers. Under controlled twist conditions, the α depends on the length of the rope. The average twist angle for the rope samples of comparable length was $\alpha = 14\pm4°$. On further twisting, the limiting $\alpha = 32°$; beyond this the rope samples spontaneously turns in the opposite direction. For rope samples having initial $\alpha = 14\pm4°$, the energy storage phenomenon does not change significantly, as for mechanical energy storage experiments, we twist the rope samples with a motor in the direction of their initial twist.

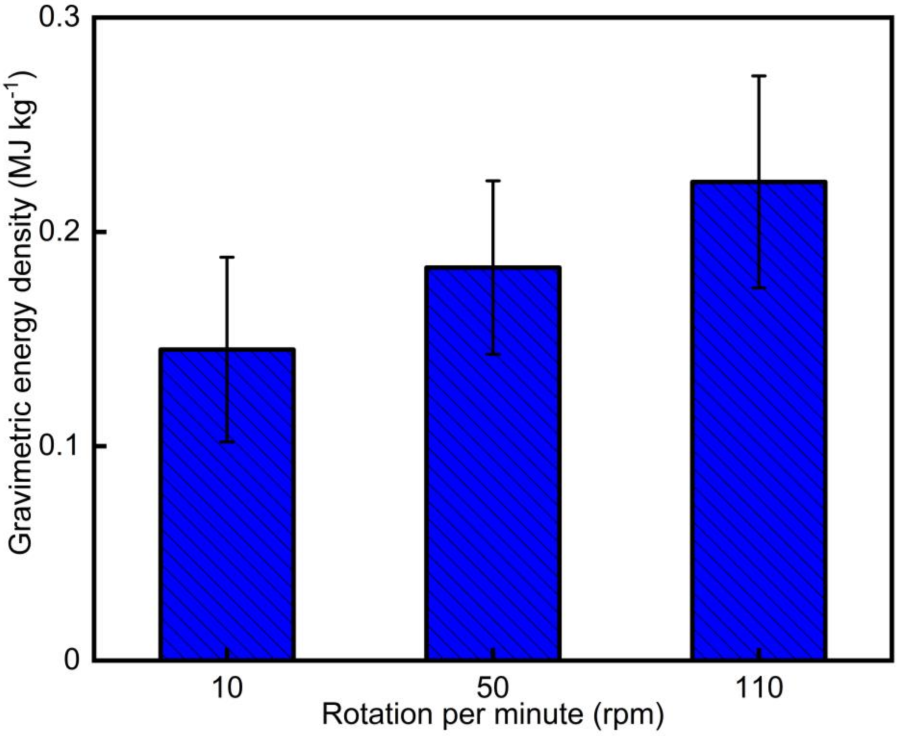

Fig. 25. Twist speed dependence of GED. The GED for y-rope is ≈ 35% higher at 110 rotations per minute (rpm) compared to the one twisted at 10 rpm. The data are presented as the mean ± s.d. for n = 3 measurements.

The above graph shows continuous increment in the energy storage with increase in the rotation speed. This may be associated with the structural relaxation effect in the SWCNTs bundles present in the ropes. For slower rpm, the SWCNTs bundles get sufficiently large time to attain structural relaxation, while at higher rpm the system does not get sufficient relaxation time, resulting in a 35% enhancement in the energy storage. So, all the experiments here are performed at a twisting speed 110 rpm being the maximum speed at which the rotation number could be counted by a lab-made motor equipment.
